# Functional Characterisation of the Quorum‐Sensing Regulator ExpR_Ecz_ in Modulation of *Dickeya oryzae* Motility and Virulence

**DOI:** 10.1111/mpp.70274

**Published:** 2026-06-22

**Authors:** Zhibin Liang, Huidi Liu, Ling He, Zhongqiao Chen, Weihan Gu, Xiaoyan Wu, Qunyi Chen, Xinbo Wang, Lian‐Hui Zhang

**Affiliations:** ^1^ Guangdong Provincial Key Laboratory of Microbial Signals and Disease Control, Integrative Microbiology Research Centre South China Agricultural University Guangzhou China

**Keywords:** AHL, *Dickeya oryzae*, motility, quorum sensing, virulence

## Abstract

The AHL (acyl‐homoserine lactone)‐mediated quorum‐sensing (QS) regulatory mechanism is ubiquitously distributed in gram‐negative pathogens. In *Dickeya oryzae*, *N*‐(3‐oxo‐hexanoyl)‐L‐homoserine lactone (OHHL), synthesised by ExpI_Ecz_, regulates bacterial swimming motility and virulence. However, the role of its putative cognate receptor ExpR_Ecz_ has not yet been characterised. In this study, we showed that deletion of *expR*_*Ecz*_ alone did not cause significant changes in various phenotypes associated with *D. oryzae* physiology and pathogenesis. However, in the absence of a functional OHHL synthase ExpI_Ecz_, ExpR_Ecz_ was a potent repressor modulating the production of cellulases, polygalacturonases and zeamines, biofilm formation and pathogenicity of *D. oryzae* EC1, and it was a positive regulator modulating bacterial swimming motility. Further analysis showed that the regulatory activity of ExpR_Ecz_ was abolished by OHHL, and that the QS signal might relieve the regulation of ExpR_Ecz_ by interacting with two conserved AHL‐binding residues of ExpR_Ecz_. Moreover, we found that ExpR_Ecz_ repressed its own expression through a region in the 5′ noncoding region of *expR*_*Ecz*_ that lacks a canonical *lux* box. These findings suggest that the mechanism may enable *D. oryzae* to relieve ExpR_Ecz_ suppression and initiate infection at high cell density when the QS signal reaches a threshold level.

## Graphical abstract

In the absence of OHHL synthase ExpI_Ecz_, ExpR_Ecz_ represses self‐expression via EDDR region and regulates *Dickeya oryzae* virulence traits. OHHL synthesised via ExpI_Ecz_ abolishes ExpR_Ecz_ function but promotes OHHL production and *D. oryzae* virulence.

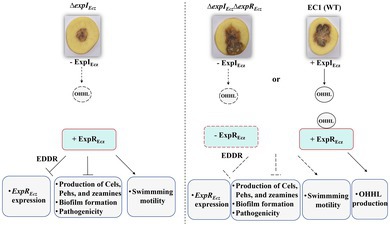

## 1 Introduction

*Dickeya* is recognised as one of the top 10 important bacterial phytopathogens globally (Mansfield et al. 2012), with members of the genus capable of infecting potato, rice, banana and a range of other crops and plants (Hu et al. 2018; Samson et al. 2005; van der Wolf et al. 2014; Wang et al. 2020). *Dickeya oryzae* is an important bacterial pathogen separated from *Dickeya zeae* recently (Wang et al. 2020), which causes soft rot diseases in rice and potato, resulting in significant economic losses across Asia and Europe (Bertani et al. 2013; Liu et al. 2013; Pritchard et al. 2013). The pathogen relies on a range virulence‐associated factors such as phytotoxin zeamines (Zhou et al. 2011; Cheng et al. 2013), cell wall‐degrading enzymes (CWDEs), bacterial motility and biofilm formation (Hussain et al. 2008; Chen et al. 2020; Shi et al. 2019; Lv et al. 2018), which collectively contribute to its pathogenicity and virulence.

Quorum sensing (QS) is a common regulatory mechanism in bacteria responsible for regulation of diverse phenotypes, including virulence factor production, bacterial motility and antibiotic production (Zhang 2003). *D. oryzae* employs at least three QS systems, that is, AHL (acyl‐homoserine lactone), VFM (virulence factor modulating) and PUT (putrescine), to modulate virulence factor production and pathogenicity against both monocot and dicot plants (Hussain et al. 2008; Lv et al. 2019; Shi et al. 2019). Among them, the AHL‐type signal *N*‐(3‐oxo‐hexanoyl)‐L‐homoserine lactone (OHHL) plays a significant role in modulation of bacterial swimming motility, biofilm formation and production of CWDEs. Deletion of *expI*_*Ecz*_, which encodes an enzyme responsible for OHHL biosynthesis, enhances bacterial motility (Hussain et al. 2008; Zhou et al. 2016). In contrast, its putative cognate regulator ExpR_Ecz_ remains uncharacterised.

The findings on the roles and regulatory mechanisms of AHL QS systems in different phytopathogens highlight both conservation and variations in regulatory mechanisms. In the canonical AHL‐type QS system, the AHL signal binds and activates its receptor LuxR, and consequently upregulates the expression of *luxI*/*luxR* and downstream target genes at a high cell density (Zhang 2003). However, the LuxR homologues in soft rot pathogens, such as ExpR_Ecc_ in *Erwinia carotovora* , ExpR in *Dickeya dandatii* and EsaR in *Pantoea stewartii* subsp. *stewartii*, do not regulate the expression of *lux* homologous genes (Nasser et al. 1998; Andersson et al. 2000). Interestingly, inactivation of *expR*_*Ecc*_ in *E. carotovora* increased AHL signal production (Andersson et al. 2000). Similarly, in *D. dandatii* and *P. stewartii* subsp. *stewartii*, bacterial cells could still produce AHL‐type signals in the absence of ExpR or EsaR (Minogue et al. 2002; Reverchon et al. 1998). Further study showed that EsaR of *P. stewartii* subsp. *stewartii* acts as a negative regulator that represses the expression of its coding gene and the genes controlling extracellular polysaccharide production. When the bacteria reach a high cell density, the accumulated QS OHHL signal molecules interact with EsaR and release its repression on the target genes (Minogue et al. 2002; Minogue et al. 2005; Carlier and von Bodman 2006; Roper 2011). Similarly, in *D. dandatii* 3937 (previously named as *Erwinia chrysanthemi* 3937), the OHHL signal binds to the LuxR homologue ExpR, and consequently derepresses the transcription of its coding gene *expR*. Expression of *expR* is dependent on bacterial growth and reaches a peak level at the exponential stage (Castang et al. 2006; Nasser et al. 1998). In *E. carotovora* , inactivation of *expR*_*Ecc*_ slightly increases Pel production (Andersson et al. 2000). It is not yet clear how ExpR_Ecz_ in *D. oryzae* may act in the regulation of the bacterial physiology and pathogenesis.

In this study, to determine the role of ExpR_Ecz_ in *D. oryzae* , in‐frame deletion of *expR*_*Ecz*_ was performed on both the wild‐type strain EC1 and the *expI*_*Ecz*_ gene mutant Δ*expI*_*Ecz*_ to elucidate whether *expI*_*Ecz*_‐dependent repression of the bacterial motility requires a functional ExpR_Ecz_. The results indicate that deletion of *expR*_*Ecz*_ in the mutant Δ*expI*_*Ecz*_ restored the bacterial swimming motility, production of cellulases, polygalacturonases and zeamines, and virulence to the wild‐type level. In addition, ExpR_Ecz_ negatively autoregulated its own expression, and OHHL abolished this autoregulation. Two conserved amino acid residues in the AHL‐binding domain of ExpR_Ecz_ were found to be essential for the ExpR_Ecz_‐mediated regulation of the bacterial motility. These findings add to our understanding of the roles and regulatory mechanism of the AHL‐type QS system in *D. oryzae* .

## 2 Results

### 2.1 ExpR_Ecz_ Null Mutation Affects AHL Biosynthesis

To determine the potential function and regulatory mechanism of ExpR_Ecz_ in *D. oryzae* EC1, sequence alignment was performed among the amino acid sequences of ExpR_Ecz_, LuxR (Vannini et al. 2002) and four well‐studied LuxR homologues, that is, TraR (Luo et al. 2003), LasR (Bottomley et al. 2007), EsaR (Minogue et al. 2002) and ExpR (Vannini et al. 2002), to identify the conserved residues putatively associated with OHHL binding. The results indicate that the residues Y50 and W54 of ExpR_Ecz_ aligned well with the Y53 and W57 of TraR, Y56 and W60 of LasR, and Y62 and W66 of LuxR (Figure S1A), which are known to interact with the homoserine lactone moiety by hydrogen bonding (Vannini et al. 2002; Luo et al. 2003; Churchill and Chen 2011), suggesting that ExpR_Ecz_ could physically interact with the AHL signal. Interestingly, phylogenetic analysis showed that ExpR_Ecz_ is clustered with ExpR_Ecc_ and EsaR, but separated from those using AHL as an activator ligand, including TraR, LasR and LuxR (Figure S1B). Together, these findings suggest that, similar to EsaR (Minogue et al. 2002), ExpR_Ecz_ might act as a repressor of AHL biosynthesis and its repressor activity could be inactivated by the cognate AHL signal.

**FIGURE 1:**
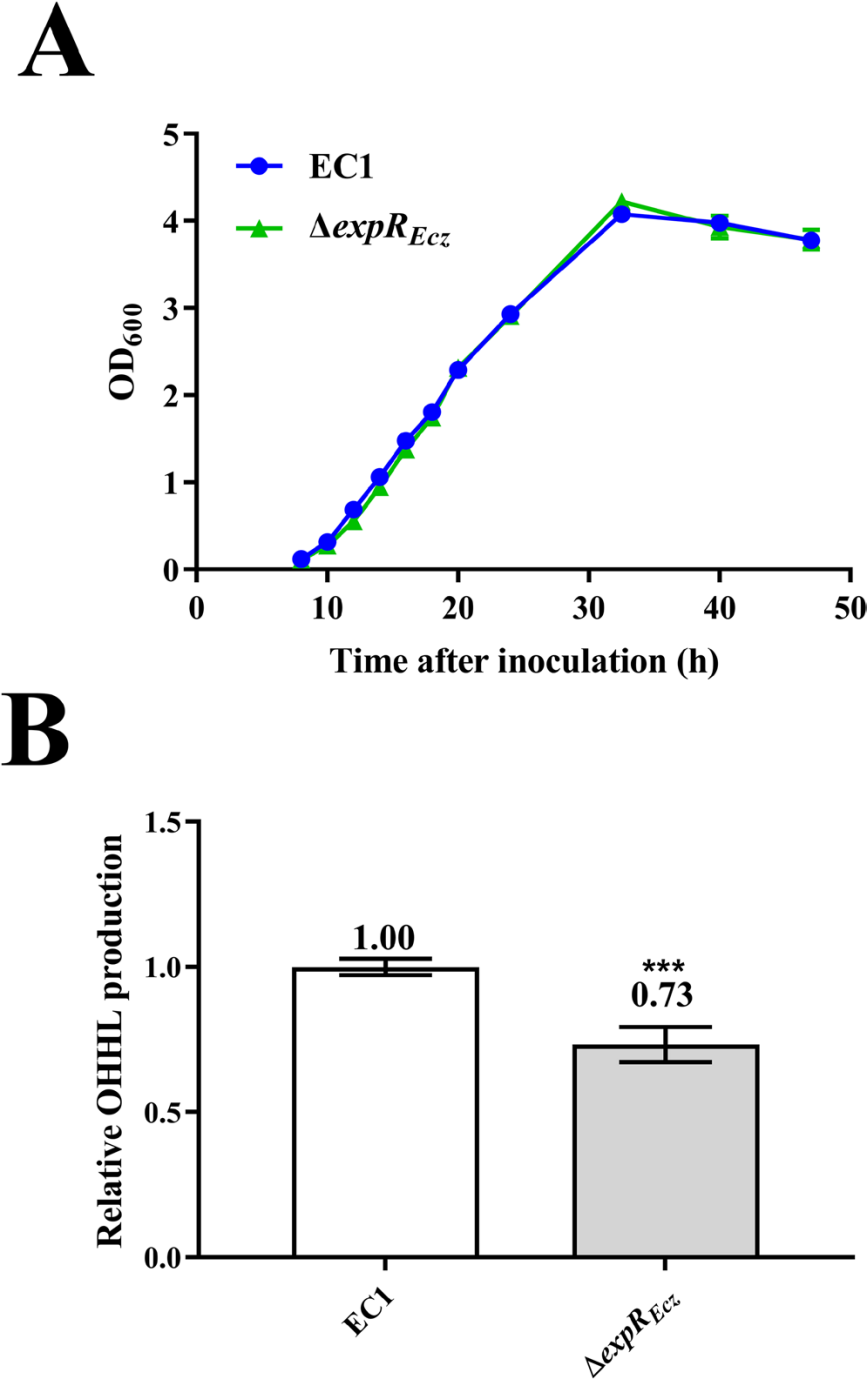
Effect of ExpR_Ecz_ on bacterial growth and OHHL production in *Dickeya oryzae* EC1. (A) Growth patterns of strain EC1 and mutant Δ*expR*_*Ecz*_. Data are presented as mean ± SD, *n* = 3. (B) OHHL production determined by UPLC‐MS. The amount of OHHL produced by strain EC1 served as the control and was set as 1 for the convenience of comparison. Data are presented as mean ± SD, *n* = 6. Statistical analysis was performed using a two‐tailed unpaired Student's *t*‐test, ***p < 0.001.

To validate the above findings, in‐frame deletion of *expR*_*Ecz*_ was performed in the wild‐type strain EC1 to construct the *expR*_*Ecz*_ mutant Δ*expR*_*Ecz*_. Bacterial growth of Δ*expR*_*Ecz*_ was determined in LS5 medium, which is a minimal medium optimised for zeamine production (Liao et al. 2014). The results showed that the mutant Δ*expR*_*Ecz*_ has a comparable growth pattern to the wild‐type strain EC1 (Figure 1A), indicating that ExpR_Ecz_ does not regulate the growth of *D. oryzae* . OHHL production was then determined by diffusion plate assay using a cell culture of Δ*expR*_*Ecz*_ and the AHL biosensor *Agrobacterium tumefaciens* CF11, which turns blue in the presence of AHL signals. The wild‐type strain EC1 was used for comparison, and the *expI*_*Ecz*_ deletion mutant Δ*expI*_*Ecz*_ was used as the negative control. Contrary to the phenotype of the mutant Δ*expI*_*Ecz*_, deletion of *expR*_*Ecz*_ did not abolish OHHL production, but appeared to produce a lesser amount of OHHL than the wild‐type strain EC1 (Figure S2A). OHHL production of Δ*expR*_*Ecz*_ was further determined by extracting OHHL from cell‐free supernatant followed by semi‐quantification using an assay by ultraperformance liquid chromatography coupled with mass spectrometry (UPLC‐MS). The results showed that inactivation of *expR*_*Ecz*_ led to about a 27% decrease in OHHL production compared to its parental strain EC1 (Figure 1B and Figure S2B), suggesting that ExpR_Ecz_ is partially associated with the positive regulation of OHHL production in *D. oryzae* .

### 2.2 ExpR_Ecz_ Null Mutation in Mutant Δ*expI*_*Ecz*_ Rescues Its Cellulase and Polygalacturonase Production to the Wild‐Type Level

**FIGURE 2:**
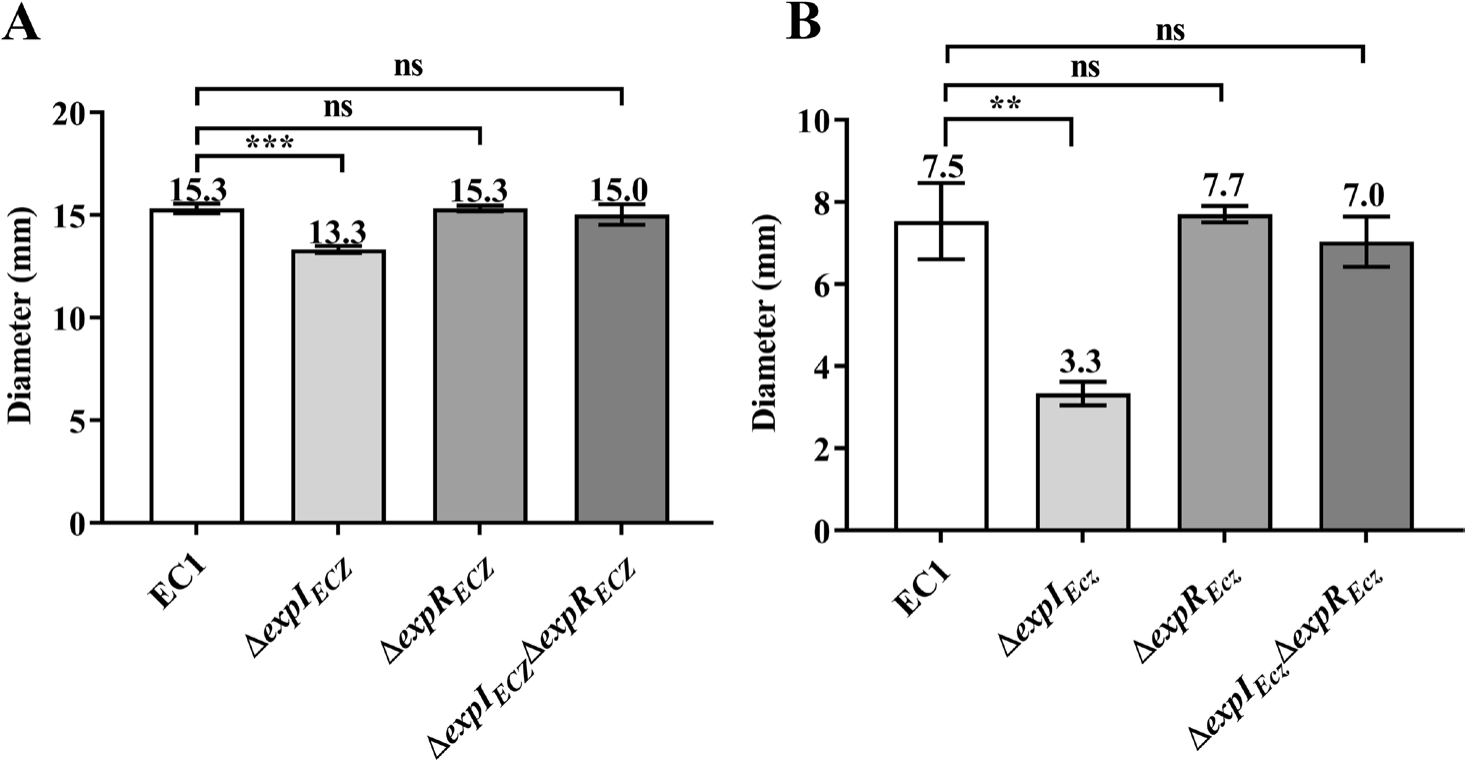
Qualitative analysis of cellulase and polygalacturonase production of strain EC1 and its derivatives. Strain EC1 and its derivatives were cultured in LS5 medium. The cell cultures of strain EC1 and its derivatives were inoculated to the wells in the cellulase (A) and polygalacturonase (B) assay plates. Data are presented as mean ± SD, *n* = 3. Statistical analysis was performed using a two‐tailed unpaired Student's *t*‐test, **p < 0.01, ***p < 0.001; ns, not significant.

Cellulase (Cel) and polygalacturonase (Peh) are two types of CWDEs produced by *D. oryzae* (Lv et al. 2022). To determine the role of ExpR_Ecz_ in regulation of Cel and Peh production, bacterial cells were cultured in LS5 medium overnight, and the cell cultures were adjusted to an OD_600_ of about 1.0 prior to inoculation into Cel and Peh assay plates. The results showed that unlike mutant Δ*expI*_*Ecz*_, which produced a lesser amount of Cels and Pehs, the mutant Δ*expR*_*Ecz*_ produced a comparable level of both enzymes as the wild‐type strain EC1 (Figure 2 and Figure S3). Interestingly, however, in‐frame deletion of *expR*_*Ecz*_ in Δ*expI*_*Ecz*_ restored Cel and Peh production of Δ*expI*_*Ecz*_ to the wild‐type level (Figure 2 and Figure S3), suggesting that ExpR_Ecz_ plays a negative role in modulation of Cel and Peh production in mutant Δ*expI*_*Ecz*_, but appeared neutral in the wild‐type strain EC1.

### 2.3 ExpR_Ecz_ Null Mutation in Mutant Δ*expI*_*Ecz*_ Rescues Its Phytotoxin Production to the Wild‐Type Level

**FIGURE 3:**
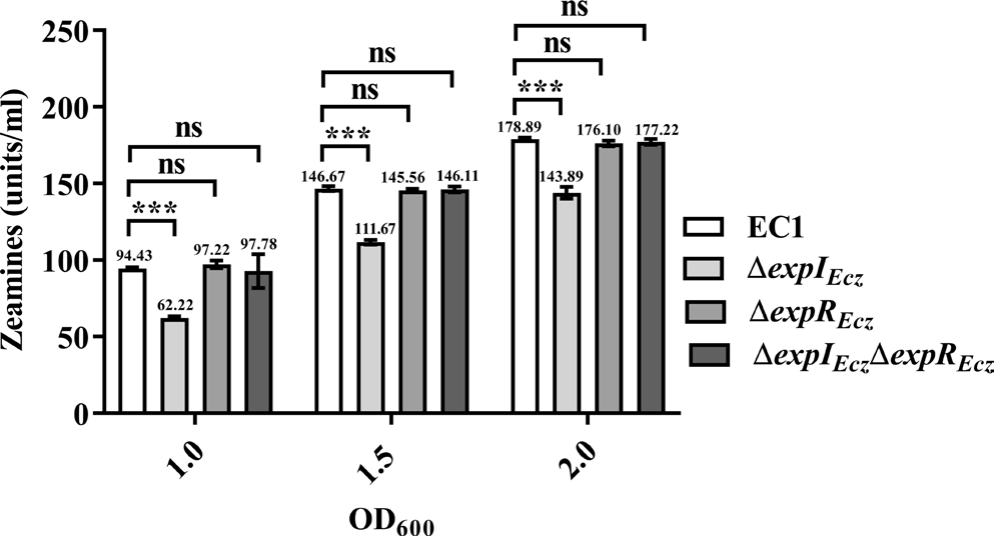
Qualitative analysis of zeamine production of strain EC1 and its derivatives. The cell‐free supernatants of strain EC1 and its derivatives cultured in LS5 medium were collected and sterilised by filtration for zeamine production assay. Data are presented as mean ± SD, *n* = 3. Statistical analysis was performed using a two‐tailed unpaired Student's *t*‐test at different cell densities, ***p < 0.001; ns, not significant.

Zeamines are the phytotoxins produced by *D. oryzae* EC1 that can inhibit rice seed germination and contribute to the virulence of *D. oryzae* EC1 (Zhou et al. 2011; Cheng et al. 2013). In this study, we found that mutant Δ*expI*_*Ecz*_ produced a significantly less amount of zeamines, but zeamine production of mutants Δ*expR*_*Ecz*_ and Δ*expI*_*Ecz*_Δ*expR*_*Ecz*_ was comparable to that of the wild‐type strain EC1 (Figure 3), suggesting that ExpR_Ecz_ plays a negative role in the regulation of zeamine production in mutant *expI*_*Ecz*_ but not in the wild‐type strain EC1.

### 2.4 ExpR_Ecz_ Null Mutation in Mutant Δ*expI*_*Ecz*_ Rescues Its Motility and Biofilm Formation to the Wild‐Type Level

**FIGURE 4:**
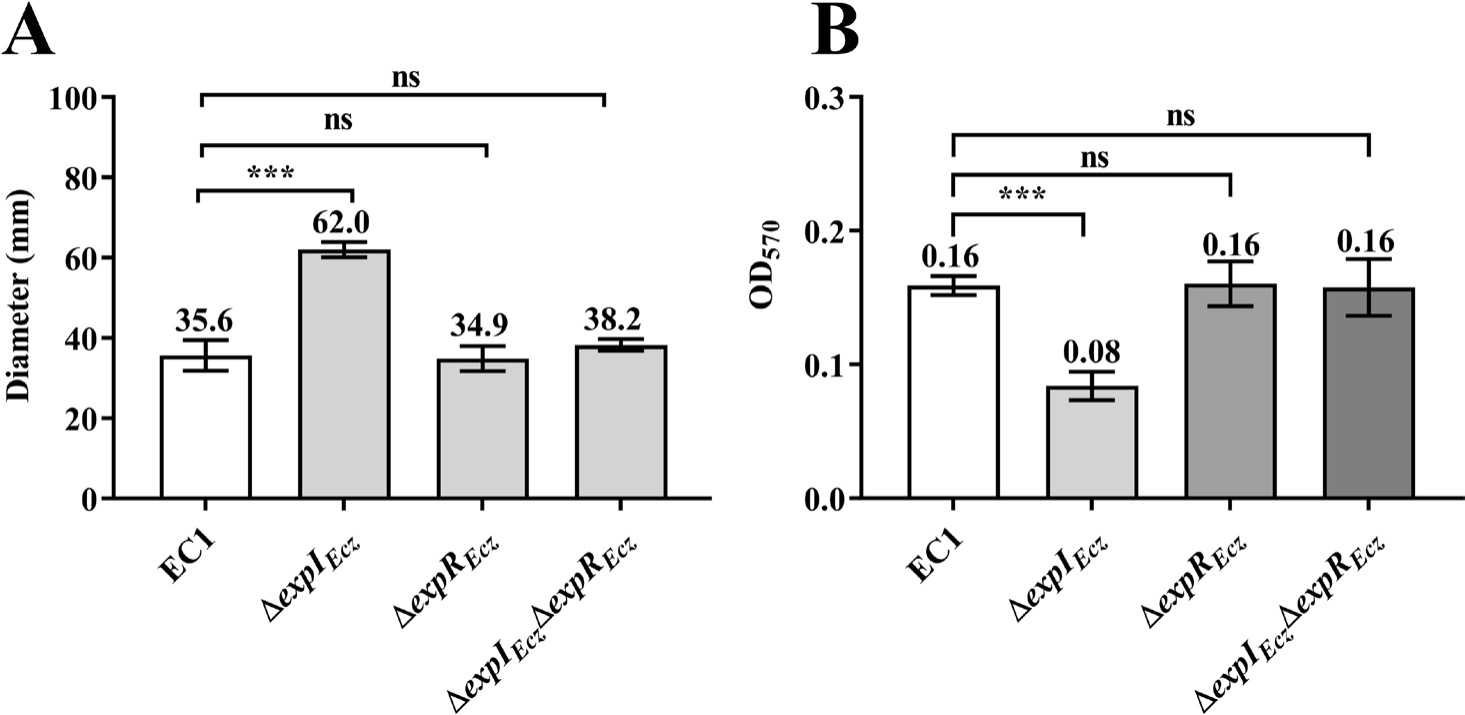
Qualitative analysis of swimming motility and biofilm formation of strain EC1 and its derivatives. (A) Swimming motility of strain EC1 and its derivatives was assayed in the semisolid agar plates, and the diameter of chemotactic zones was measured. Data are presented as mean ± SD, *n* = 3. (B) Biofilm formation of strain EC1 and its derivatives measured by using glass tubes. Statistical analysis was performed using a two‐tailed unpaired Student's *t*‐test, ***p < 0.001; ns, not significant.

Our previous study showed that inactivation of *expI*_*Ecz*_ significantly enhances the swimming motility of *D. oryzae* (Hussain et al. 2008). In contrast, inactivation of *expR*_*Ecz*_ did not affect the swimming motility of the wild‐type strain EC1 (Figure 4A and Figure S4A). To determine whether increased swimming motility of mutant Δ*expI*_*Ecz*_ was also dependent on ExpR_Ecz_, the swimming motility of Δ*expI*_*Ecz*_, Δ*expR*_*Ecz*_ and Δ*expI*_*Ecz*_Δ*expR*_*Ecz*_ were compared with the wild‐type strain EC1. The results showed that deletion of *expI*_*Ecz*_ increased *D. oryzae* swimming motility (Figure 4A and Figure S4A), and exogenous addition of OHHL restored the swimming motility of the mutant Δ*expI*_*Ecz*_ to the wild‐type level (Figure S5A). Double‐deletion mutant Δ*expI*_*Ecz*_Δ*expR*_*Ecz*_ showed a comparable swimming motility to the wild‐type strain EC1 and mutant Δ*expR*_*Ecz*_ (Figure 4A and Figure S4A), suggesting that ExpR_Ecz_ positively regulates the swimming motility of the *expI*_*Ecz*_ deletion mutant. Consistent with the above findings, inactivation of *expR*_*Ecz*_ did not generate any obvious impact on biofilm formation compared with the wild‐type strain EC1, but deletion of *expR*_*Ecz*_ in the mutant Δ*expI*_*Ecz*_ rescued the biofilm formation defect to the wild‐type level (Figure 4B and Figure S4B), suggesting that ExpR_Ecz_ plays a negative role in modulation of biofilm formation in the *expI*_*Ecz*_ deletion mutant.

### 2.5 ExpR_Ecz_ Null Mutation Rescues the Virulence of *expI*_*Ecz*_ Mutant to the Wild‐Type Level

**FIGURE 5:**
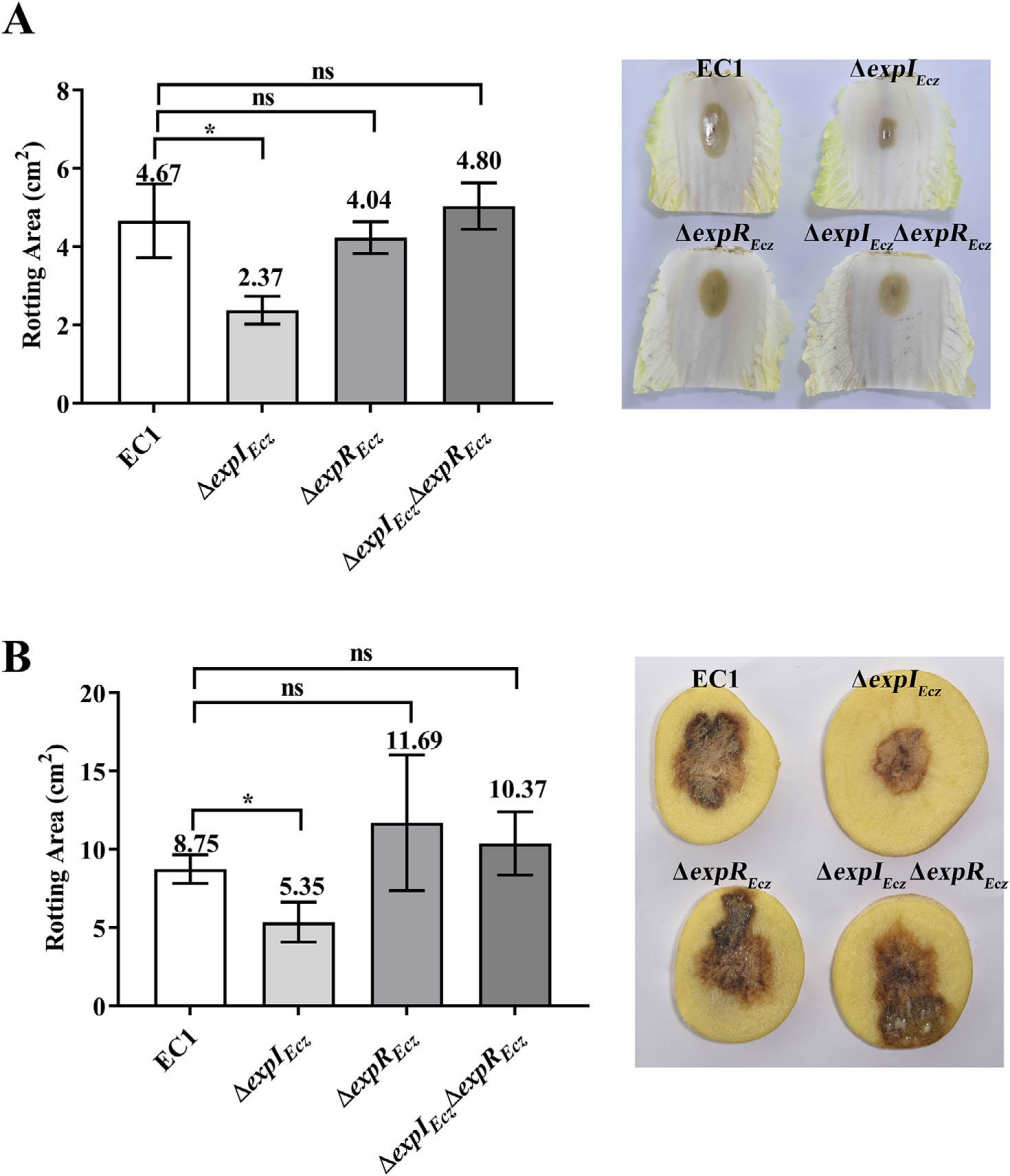
Virulence of strain EC1 and its derivatives against Chinese cabbage leaves and potato tubers. Strain EC1 and its derivatives were inoculated into sliced Chinese cabbage leaves (A) or potato tubers (B). The rotting areas were measured at 48 h post‐inoculation. Data are presented as mean ± SD, *n* = 4. Statistical analysis was performed using the Mann–Whitney test, *p < 0.05; ns, not significant.

To determine the role of ExpR_Ecz_ in *D. oryzae* virulence, wild‐type strain EC1 and its derivatives were inoculated in Chinese cabbage leaves and potato tubers. As reported previously (Hussain et al. 2008), the *expI*_*Ecz*_ mutant showed significantly attenuated virulence against both plants (Figure 5). Consistent with the role of ExpI_Ecz_ in biosynthesis of AHL signals, exogenous addition of OHHL restored the virulence of the *expI*_*Ecz*_ mutant (Figure S5B,C). In contrast, inactivation of *expR*_*Ecz*_ in the wild‐type strain EC1 did not affect the virulence of *D. oryzae* EC1 on Chinese cabbage leaves and potato tubers, whereas its deletion in the mutant Δ*expI*_*Ecz*_ restored the virulence to the wild‐type level (Figure 5). These results suggest that ExpR_Ecz_ negatively regulates bacterial virulence in the absence of ExpI_Ecz_ but not in the wild‐type strain EC1.

### 2.6 ExpR_Ecz_ Negatively Regulates Its Own Transcriptional Expression

**FIGURE 6:**
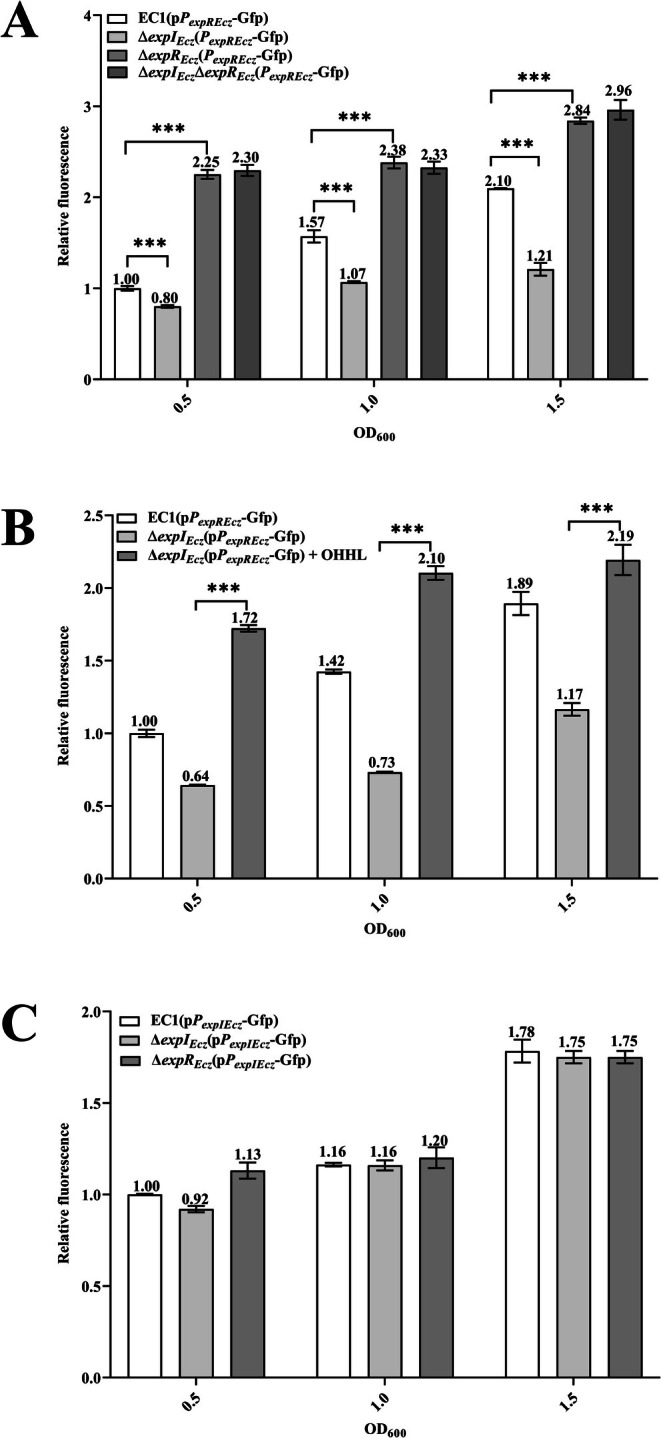
Regulation of ExpI_Ecz_ /ExpR_Ecz_ quorum‐sensing system on *expI*_*Ecz*_ and *expR*_*Ecz*_ expression. (A) Relative fluorescence of strain EC1(p*P*_*expREcz*_‐Gfp), ∆*expI*_*Ecz*_(p*P*_*expREcz*_‐Gfp), ∆*expR*_*Ecz*_(p*P*_*expREcz*_‐Gfp) and ∆*expI*_*Ecz*_∆*expR*_*Ecz*_(p*P*_*expREcz*_‐Gfp) at different cell densities. (B) Relative fluorescence of EC1(p*P*_*expREcz*_‐Gfp), ∆*expI*_*Ecz*_(p*P*_*expREcz*_‐Gfp) and ∆*expI*_*Ecz*_(p*P*_*expREcz*_‐Gfp) in presence of OHHL (2 μM), at different cell densities. (C) Relative fluorescence of EC1(p*P*_*expIEcz*_‐Gfp), ∆*expI*_*Ecz*_(p*P*_*expIEcz*_‐Gfp) and ∆*expR*_*Ecz*_(p*P*_*expIEcz*_‐Gfp) at different cell densities. Data are presented as mean ± SD, *n* = 3. The value of strain EC1(p*P*_*expREcz*_‐Gfp) (A and B) or EC1(p*P*_*expIEcz*_‐Gfp) (C) at the cell density at OD_600_ about 0.5 was conducted as the control and set as 1 for normalisation. Statistical analysis was performed using a two‐tailed unpaired Student's *t*‐test, ***p < 0.001.

Previous works documented the canonical regulatory mechanism of AHL‐type QS system, in which expression of *luxI* and *luxR* is triggered by the AHL‐LuxR complex at high cell density (Zhang 2003). However, an exception was found in *E. carotovora* , in which ExpR_Ecc_ does not affect *expI* transcription (Andersson et al. 2000). To elucidate whether the *expI*_*Ecz*_ and *expR*_*Ecz*_ genes are autoregulated in *D. oryzae* EC1, an approximately 250‐bp 5′ noncoding region of *expI*_*Ecz*_ (*P*_*expIEcz*_, 266 bp) and *expR*_*Ecz*_ (*P*_*expREcz*_, 245 bp) containing putative −35 and −10 elements were used for constructing the reporter gene constructs p*P*_*expIEcz*_‐Gfp and p*P*_*expREcz*_‐Gfp, respectively. These reporter constructs were introduced into the wild‐type strain EC1 and derivatives, and the *expI*_*Ecz*_‐ or *expR*_*Ecz*_‐promoter‐directed *gfp* expression was measured accordingly. Firstly, we checked the *expR*_*Ecz*_ expression in the wild‐type strain EC1 and its derivatives. The results showed that the relative fluorescence of strain EC1(p*P*_*expREcz*_‐Gfp) increased along with the bacterial growth, which was higher than that of mutant ∆*expI*_*Ecz*_(p*P*_*expREcz*_‐Gfp) but lower than that of mutant ∆*expR*_*Ecz*_(p*P*_*expREcz*_‐Gfp) at different bacterial growth stages (Figure 6A), indicating that ExpR_Ecz_ is synthesised in a growth‐dependent manner (Figure 6B), and ExpI_Ecz_ and ExpR_Ecz_ exerted opposite effects on the transcriptional expression of *expR*_*Ecz*_. Considering that the product of ExpI_Ecz_ is the QS signal OHHL, we then determined the impact of exogenous addition of OHHL on *expR*_*Ecz*_ expression in the mutant ∆*expI*_*Ecz*_. The results showed that exogenous addition of OHHL significantly increased the relative fluorescence of mutant ∆*expI*_*Ecz*_(p*P*_*expREcz*_‐Gfp) (Figure 6B), suggesting that the OHHL produced by ExpI_Ecz_ could relieve ExR_Ecz_ repression. We further compared the *expI*_*Ecz*_ expression in strain EC1 and its derivatives. We found that expression of *expI*_*Ecz*_ was elevated at the late growth stage (Figure 6C), and unlike the significant influence of ExpI_Ecz_ or ExpR_Ecz_ on *expR*_*Ecz*_ expression, we found that null mutation of ExpI_Ecz_ or ExpR_Ecz_ did not generate a significant impact on *expI*_*Ec*_ expression, as the relative fluorescence of mutant ∆*expI*_*Ecz*_(p*P*_*expIEcz*_‐Gfp) or mutant ∆*expR*_*Ecz*_(p*P*_*expIEcz*_‐Gfp) was comparable to that of strain EC1(p*P*_*expIEcz*_‐Gfp) at different bacterial growth stages (Figure 6C).

### 2.7 Identification of the Promoter Element of *expR*_*Ecz*_ Essential for ExpR_Ecz_ Self‐Regulation

**FIGURE 7:**
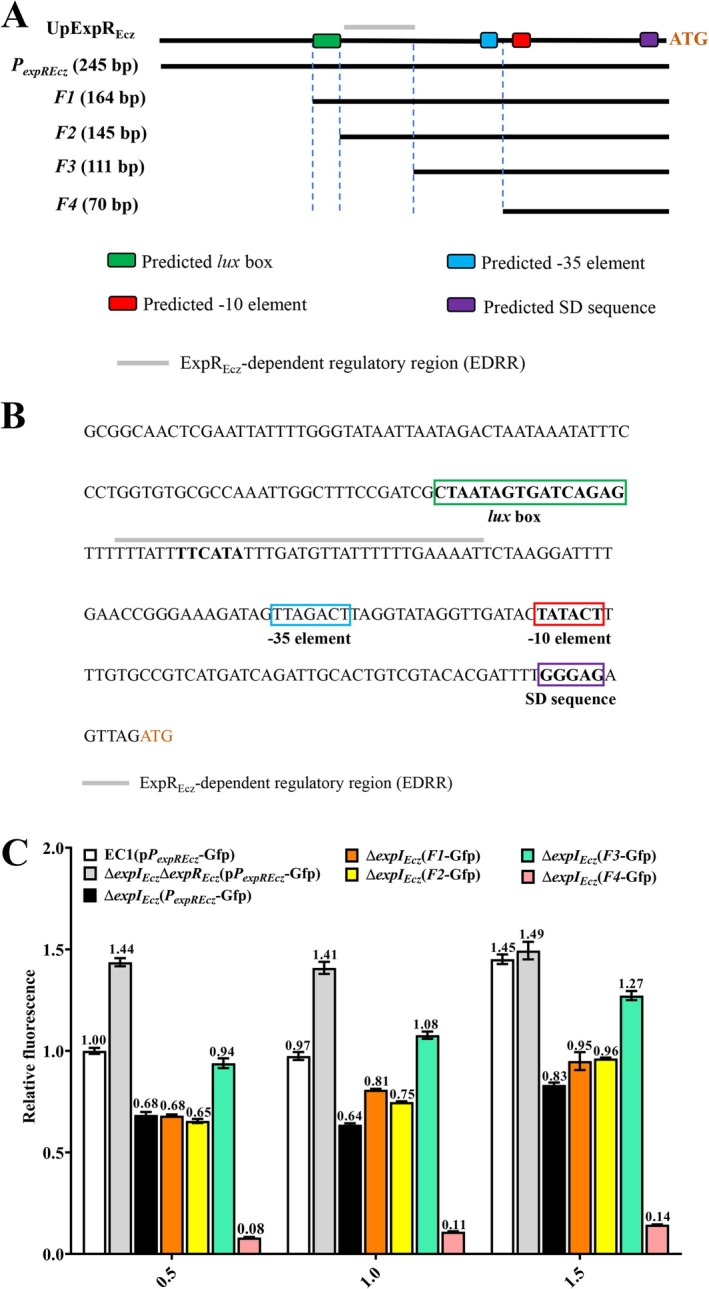
Characterisation of the 5′ noncoding region of *expR*_*Ecz*_. The 5′ noncoding region of *expR*_*Ecz*_ (*P*_*expREcz*_) with different sizes (F1–F4) were fused with the coding sequence of Gfp in the plasmid pPROBE‐NT to generate the reporter constructs *F1*‐Gfp, *F2*‐Gfp, *F3*‐Gfp and *F4*‐Gfp, respectively, to identify the essential region for autoregulation of *expR*_*Ecz*_. (A) Schematic diagram of *P*_*expREcz*_. (B) DNA sequence of *P*_*expREcz*_. The bold fonts show the *lux* box (CTAATAGTGATCAGAG), −35 and −10 elements (TTCATA and TATACT, respectively), and SD sequence (GGAG) predicted in a previous study (Hussain et al. 2008). The *lux* box, −35 and −10 elements, and SD sequence predicted in this study are indicated with green, blue, red and purple frames, respectively. The start codon of *expR*_*Ecz*_ (ATG) is also presented using orange font. (C) The relative fluorescence of the reporter strain EC1(p*P*_*expREcz*_‐Gfp), ∆*expI*_*Ecz*_∆*expR*_*Ecz*_(p*P*_*expREcz*_‐Gfp), ∆*expI*_*Ecz*_(p*P*_*expREcz*_‐Gfp), ∆*expI*_*Ecz*_(*F1*‐Gfp), ∆*expI*_*Ecz*_(*F2*‐Gfp), ∆*expI*_*Ecz*_(*F3*‐Gfp) and ∆*expI*_*Ecz*_(*F4*‐Gfp) measured at different cell densities. The relative fluorescence of EC1(p*P*_*expIEcz*_‐Gfp) at an OD_600_ about 0.5 was used as the control and set as 1 for normalisation. Data are presented as mean ± SD, *n* = 3.

Given that *expR*_*Ecz*_ expression is positively regulated by OHHL and negatively controlled by ExpR_Ecz_ (Figure 6A,B), we set out to determine the promoter elements of *expR*_*Ecz*_ essential for ExpR_Ecz_ self‐regulation in the genetic background of OHHL‐minus mutant Δ*expI*_*Ecz*_. Four truncated *P*_*expREcz*_ fragments (F1 to F4) with different sizes were PCR amplified (Figure 7A,B). F1 is a 164‐bp fragment containing the *lux* box, −35 and −10 elements, and SD sequence as previously predicted (Hussain et al. 2008). F2 is a 145‐bp fragment derived from F1 with the predicted 19 bp *lux* box being eliminated. F3 was generated by further eliminating a 34‐bp fragment from F2. Removal of a 41‐bp fragment containing the putative −35 region predicted in this study from the 5′ end of F3 resulted in the F4 fragment (Figure 7A,B). These four fragments were fused with the coding sequence of *gfp* in the plasmid pPROBE‐NT, respectively, to generate the constructs F1‐Gfp, F2‐Gfp, F3‐Gfp and F4‐Gfp. Mutant *∆expI*_*Ecz*_ containing these constructs was then assayed to determine the key regions in *P*_*expREcz*_ responsible for ExpR_Ecz_ autoregulation. As comparisons, strain EC1 and the double deletion mutant *∆expI*_*Ecz*_ *∆expR*_*Ecz*_ harbouring their parental construct p*P*_*expIEcz*_‐Gfp were also included in the experiment. The results in Figure 7C showed that the promoter fragments F1 and F2 directed fluorescence levels similar to that of ∆*expI*_*Ecz*_(p*P*_*expREcz*_‐Gfp), whereas the expression level of F3 was increased over 30%. In contrast, fragment F4, which lacks the putative −35 element predicted in this study, basically lost the promoter activity. These results suggest that the previously predicted *lux* box does not contribute to ExpR_Ecz_ self‐regulated expression, but a 34‐bp region (named as ExpR_Ecz_‐dependent regulatory region, EDRR), which is located downstream of the predicted *lux* box (Figure 7A,B), is involved in ExpR_Ecz_ autorepression.

### 2.8 Identification of the Key Amino Acid Residues Essential for ExpR_Ecz_ Activity

**FIGURE 8:**
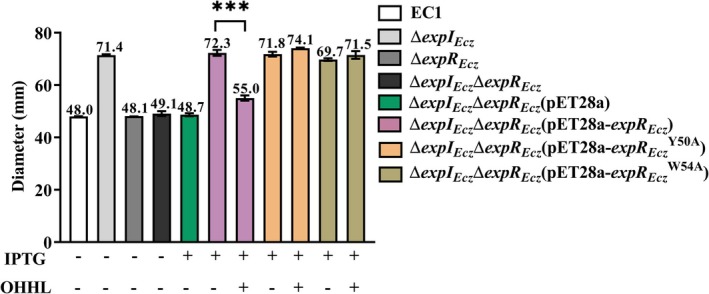
Positive regulation of ExpR_Ecz_ on bacterial swimming motility is repressed by OHHL. Swimming motility of EC1, Δ*expI*_*Ecz*_, Δ*expR*_*Ecz*_, Δ*expI*_*Ecz*_Δ*expR*_*Ecz*_ and Δ*expI*_*Ecz*_Δ*expR*_*Ecz*_ derivative strains with or without exogenous addition of IPTG (100 μM) or OHHL (2 μM). Data are presented as mean ± SD, *n* = 3. Statistical analysis was performed using a two‐tailed unpaired Student's *t*‐test, ***p < 0.001.

The previous work found that the OHHL signal synthesised by ExpI_Ecz_ plays a key role in repressing the swimming motility of *D. oryzae* EC1 (Hussain et al. 2008). To validate that whether OHHL negatively modulates *D. oryzae* swimming motility by antagonising ExpR_Ecz_ expression, the plasmid pET28a*‐expR*_*Ec*_ was constructed and transformed into the double‐deletion mutant ∆*expI*_*Ecz*_∆*expR*_*Ecz*_ to obtain strain ∆*expI*_*Ecz*_∆*expR*_*Ecz*_(pET28a*‐expR*_*Ec*_), in which expression of *expR*_*Ez*_ was induced in the presence of IPTG (isopropyl‐β‐D‐thiogalactopyranoside), under the control of the *T7lac* promoter. The results showed that induction of *expR*_*Ec*_ expression in mutant ∆*expI*_*Ecz*_∆*expR*_*Ecz*_ by IPTG led to increased bacterial swimming motility, but exogenous addition of OHHL abolished the ExpR_Ecc_‐mediated upregulation of the swimming motility of ∆*expI*_*Ecz*_∆*expR*_*Ecz*_ (Figure 8), suggesting that the OHHL signals produced by ExpI_Ecc_ could neutralise the ExpR_Ecz_‐dependent regulation of *D. oryzae* swimming motility.

To assess whether OHHL inhibits ExpR_Ecz_‐dependent regulation through the conserved AHL binding sites of ExpR_Ecz_, *expR*_*Ecz*_ derivatives were generated with alanine substitution of Y50 and W54 (Figure S1A), cloned in the expression vector pET28a, and introduced into mutant ∆*expI*_*Ecz*_∆*expR*_*Ecz*_ to construct the strains ∆*expI*_*Ecz*_∆*expR*_*Ecz*_(pET28a‐*expR*_*Ecz*_^Y50A^) and *expI*_*Ecz*_∆*expR*_*Ecz*_(pET28a‐*expR*_*Ecz*_^W54A^), respectively. The swimming motility of ∆*expI*_*Ecz*_∆*expR*_*Ecz*_(pET28a‐*expR*_*Ecz*_), ∆*expI*_*Ecz*_∆*expR*_*Ecz*_(pET28a‐*expR*_*Ecz*_^Y50A^) and *expI*_*Ecz*_∆*expR*_*Ecz*_(pET28a‐*expR*_*Ecz*_^W54A^) was assayed with or without OHHL at a final concentration of 2 μM. The results indicated that expression of either *expR*_*Ecz*_, *expR*_*Ecz*_^Y50A^ or *expR*_*Ecz*_^W54A^ in mutant ∆*expI*_*Ecz*_∆*expR*_*Ecz*_ induced by IPTG led to increased bacterial swimming motility (Figure 8), which confirmed that Y50A or W54A substitution did not affect ExpR_Ecz_ regulatory activity. However, exogenous addition of OHHL repressed the swimming motility of ∆*expI*_*Ecz*_∆*expR*_*Ecz*_(pET28a‐*expR*_*Ecz*_), but not ∆*expI*_*Ecz*_∆*expR*_*Ecz*_(pET28a‐*expR*_*Ecz*_^Y50A^) and *expI*_*Ecz*_∆*expR*_*Ecz*_(pET28a‐*expR*_*Ecz*_^W54A^) (Figure 8), suggesting OHHL might interact with the conserved AHL binding sites Y50 and W54 in ExpR_Ecz_, and thus abolish the positive regulation of ExpR_Ecz_ on the bacterial swimming motility.

## 3 Discussion

The ExpI‐ExpR‐mediated AHL QS system plays an important role in modulating the physiology and virulence traits in a range of gram‐negative bacterial pathogens. In this study, we characterised the role of ExpR_Ecz_ and its regulatory mechanism in *D. oryzae* EC1. Our results showed that in the absence of a functional ExpI_Ecz_, ExpR_Ecz_ negatively regulated Cel, Peh and zeamine production, biofilm formation, and bacterial pathogenicity (Figures 2, 3, 4B and 5, Figures S3 and S4), but positively regulated bacterial swimming motility (Figure 4A). In addition, we found that ExpR_Ecz_ repressed its own expression, mainly through a regulatory region in the 5′ noncoding region of *expR*_*Ecz*_ (Figures 6A and 7), and that OHHL abolished the autorepression of ExpR_Ecz_ (Figure 6B). Furthermore, we demonstrated that substitution of the two conserved AHL binding sites of ExpR_Ecz_ abolished the OHHL activity in antagonising the positive regulatory role of ExpR_Ecz_ on *D. oryzae* swimming motility (Figure 8).

In a typical AHL‐type QS system, the expression of the *luxI*/*luxR* gene is autoactivated by the AHL‐LuxR complex (Whiteley et al. 2017). In this study, consistent with the findings of ExpR_Ecc_, ExpR and EsaR in corresponding soft rot pathogens that do not regulate the expression of *luxI* homologues (Nasser et al. 1998; Andersson et al. 2000), we found that ExpR_Ecz_ did not modulate *expI*_*Ecz*_ expression at different growth stages of *D. oryzae* EC1 (Figure 6C). However, in contrast to the negative role of ExpR_Ecc_ in the regulation of OHHL production in *E. carotovora* (Andersson et al. 2000), our results showed that the deletion of *expR*_*Ecz*_ resulted in decreased OHHL production, indicating a positive regulatory role (Figure 1B and Figure S2). Interestingly, an ArcA binding site was found in the promoter region of *expI*_*Ecz*_ (Figure S6). Given that the two‐component system ArcBA plays a positive regulatory role in swimming motility in *D. oryzae* EC1 (Lv et al. 2022), it seems rational that *expI* expression might be regulated by ArcBA, which awaits further verification. In addition, we found that derepression of *expR*_*Ecz*_ expression was independent of cell density (Figure 7C). The above findings suggest that the regulatory pattern of the *D. oryzae* AHL QS system is in general similar to its counterparts in other soft rot pathogens, but might also differ in certain aspects, in particular, the role of ExpR_Ecz_ in modulation of QS signal production.

Similarly, conservation and variation are also obvious in the role and regulation mechanisms on the LuxR homologues in *D. oryzae* and other soft rot bacterial pathogens. Different from the sharply decreased virulence of *P. stewartii* subsp. *stewartii* EsaR mutant (von Bodman et al. 1998), deletion of ExpR_Ecz_ in *D. oryzae* EC1 did not seem to cause a statistically significant change in bacterial virulence (Figure 5). Reminiscent of ExpR and EsaR in *D. dandatii* and *P. stewartii* subsp. *stewartii*, respectively (Minogue et al. 2002; Reverchon et al. 1998), ExpR_Ecz_ acted as a negative regulator on the transcriptional expression of its coding gene (Figure 6A). We further provided evidence that the QS signal OHHL might abolish ExpR_Ecz_ regulation through direct interaction between OHHL and the conserved amino acid residues of ExpR_Ecz_, that is, Y50 and W54 (Figure S1A and Figure 8). These two amino acid residues are fully conserved in the five LuxR homologues, including ExpR_Ecc_, TraR, LasR, EsaR and LuxR (Figure S1A), and have been shown to be involved in formation of hydrogen bonds with AHL signals at least in TraR and LasR (Vannini et al. 2002; Luo et al. 2003; Churchill and Chen 2011). Notably, the corresponding resides in ExpR_Ecc_ are H50 and W54 (Figure S1A). Although H50 (a histidine residue) is not considered highly similar to the conserved Y50 (a tyrosine residue) of other LuxR homologues, it can form a hydrogen bond with AHL signals via its imidazole group.

LuxR homologue binds to the *lux* box (NNCT‐(N)_12_‐AGNN) and activates *luxI*/*luxR* expression in gram‐negative bacteria (Whiteley and Greenberg 2001; Li et al. 2022). Our previous study identified a putative *lux* box (CT‐(N)_12_‐AG) in the 5′ noncoding region of *expR*_*Ecz*_ (Figure 7A,B) (Hussain et al. 2008). However, we found that this putative *lux* box was irrelevant to ExpR_Ecz_ autorepression (Figure 7). Instead, a short sequence named EDRR in this study, without a typical *lux* box feature, was found to contribute to ExpR_Ecz_ autorepression (Figure 7). The findings seem to suggest a possibility that, unlike a canonical AHL‐type QS system, ExpR_Ecz_ may indirectly regulate its own expression, which is worthy of further investigations. Significantly, sequence alignment of the *expR*_*Ecz*_ promoter regions showed that the EDRR sequence appeared highly conserved in the related *Dickeya* species, including *D. ananatis*, *D. zeae* , *D. fangzhongdai*, *D. solani*, *D. dianthicola* and *D. dadantii* (Figure S7), suggesting a conserved and distinctive mechanism of regulation.

**FIGURE 9:**
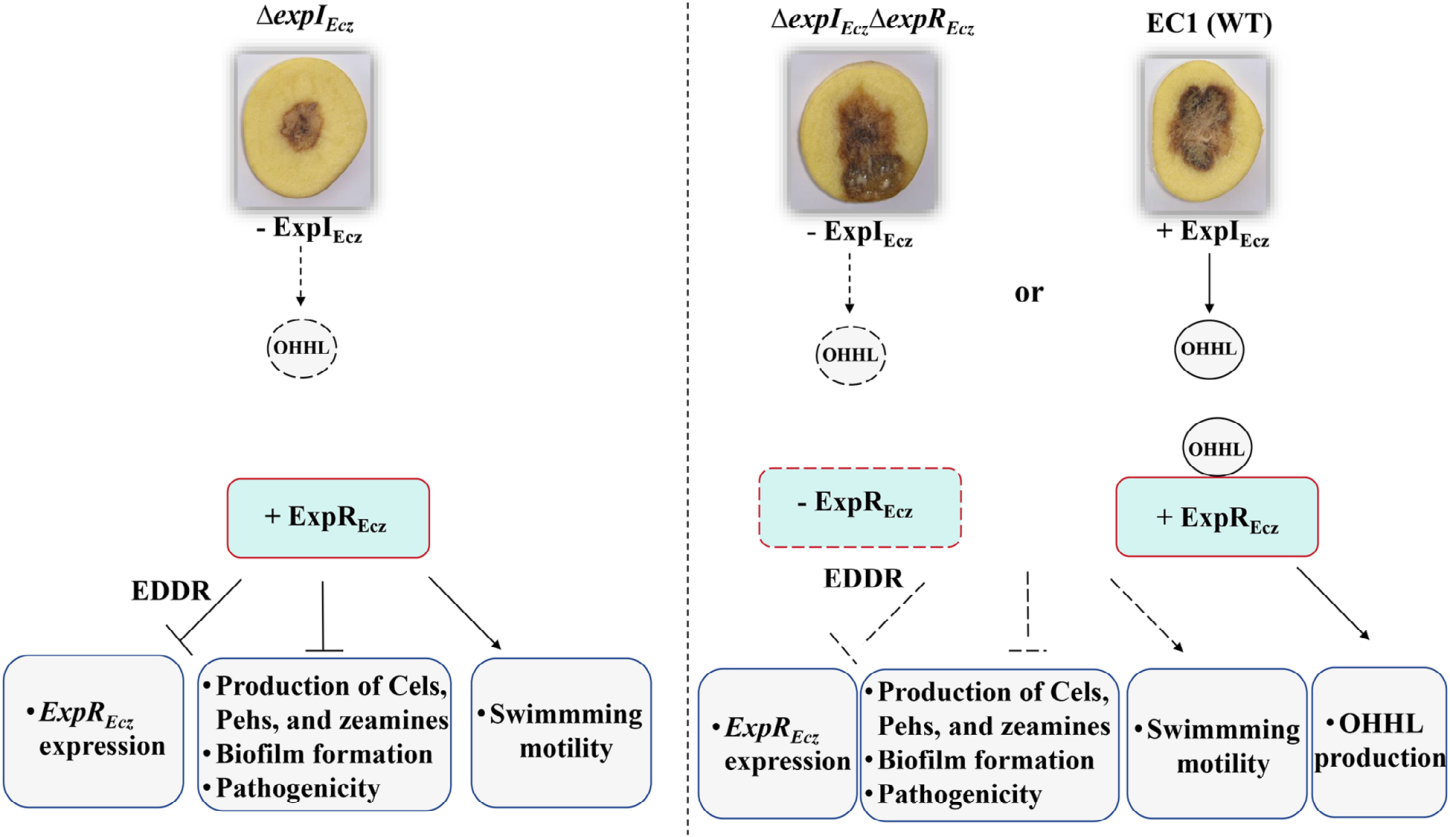
The working model of ExpR_Ecz_ in *Dickeya oryzae*. In the absence of a functional OHHL synthase ExpI_Ecz_, ExpR_Ecz_ represses its own expression through a conserved ExpR_Exz_‐dependent regulatory region (EDDR) and regulates the virulence traits and virulence of *Dickeya oryzae*. OHHL synthesised via ExpI_Ecz_ abolishes ExpR_Ecz_ function but promotes OHHL production and the virulence of *D. oryzae* .

In summary, we demonstrated that, unlike a typical AHL‐type QS system, ExpR_Ecz_ could regulate the virulence traits and bacterial virulence of *D. oryzae* EC1 in the absence of the QS signal OHHL synthesised by ExpI_Ecz_. ExpR_Ecz_ was a potent repressor of production of most virulence factors, including Cels, Pehs and zeamines, as well as biofilm formation, but not of bacterial motility in which ExpR_Ecz_ acted as a positive regulator. The virulence repression mediated by ExpR_Ecz_ was decoupled by the cognate QS signal OHHL, which might provide the bacterial pathogen a prevailing mechanism to initiate infection only at a high cell density. Interestingly, we also identified a conserved region (EDRR) essential for ExpR_Ecz_ autoregulation of its gene expression and positive regulation of OHHL production in *D. oryzae* (Figure 9). Taken together, the findings from this study unveiled the roles and mechanisms of the QS regulator ExpR_Ecz_ in modulation of *D. oryzae* physiology and virulence, and provided useful clues for further dissecting the putatively conserved autoregulation mechanism of ExpR in *Dickeya* species.

## 4 Experimental Procedures

### 4.1 Bacterial Strains, Plasmids, Primers and Growth Conditions

*Dickeya oryzae* and *Escherichia coli* derivatives used in this study are listed in Table S1. Primers used in this study are listed in Table S2. *D. oryzae* derivatives were grown at 28°C in Luria Bertani (LB), minimal medium (MM) or LS5 medium (Liang, Lin, et al. 2023). *E. coli* derivatives were grown in 37°C in LB medium. The following antibiotics were added when necessary: ampicillin, 50 μg/mL; kanamycin, 50 μg/mL; streptomycin, 50 μg/mL; polymyxin B, 50 μg/mL.

### 4.2 In‐Frame Deletion, Complementation and Point Mutation

In‐frame deletion and complementation were performed following the method described previously (Liang, Lin, et al. 2023). Briefly, for gene in‐frame deletion, suicide plasmids constructed from pKNG101 with the flanking region of *expI*_*Ecz*_ or *expR*_*Ecz*_ were transformed into the wild‐type strain EC1 or mutant ∆*expI*_*Ecz*_ by triparental mating. Desired in‐frame deletion mutants were screened on MM agar plates with 5% (wt/vol) sucrose and confirmed by PCR and DNA sequencing. For constructing complementation strains, the *expR*_*Ecz*_ gene was cloned into pET28a and expressed under the control of T7*lac* promoter in presence of IPTG (100 μM). The resultant construct pET28a‐*expR*_*Ecz*_ was transformed into ∆*expI*_*Ecz*_∆*expR*_*Ecz*_, and the complementation strain ∆*expI*_*Ecz*_∆*expR*_*Ecz*_(pET28a‐*expR*_*Ecz*_) was screened on MM agar plate with ampicillin and verified by PCR. Construction of the ∆*expI*_*Ecz*_∆*expR*_*Ecz*_(pET28a‐*expR*_*Ecz*_^Y50A^) and ∆*expI*_*Ecz*_∆*expR*_*Ecz*_(pET28a‐*expR*_*Ecz*_^W54A^) that could express ExpR_Ecz_ derivatives with alanine substition on the residue Y50 or W54 was constructed by transformation of pET28a‐*expR*_*Ecz*_^Y50A^ and pET28a‐*expR*_*EczY*_^W54A^ into ∆*expI*_*Ecz*_∆*expR*_*Ecz*_.

### 4.3 Growth Patterns

Bacterial growth patterns in LS5 medium were measured by following a previously described method (Liang et al. 2019). Briefly, overnight bacterial cell cultures grown in LB medium were inoculated to at a ratio of 0.1% (vol/vol) to the LS5 medium in the flasks. The flasks were constantly shaken at 150 rpm and 28°C. Cell density (OD_600_) was measured at different time points as indicated.

### 4.4 OHHL Production

The wild‐type strain EC1 and ∆*expR*_*Ecz*_ were grown in LS5 medium at 28°C to an OD_600_ about 1.0. About 35 mL cell‐free supernatants of EC1 and ∆*expR*_*Ecz*_ were prepared by centrifugation and filter sterilisation, and extracted twice with the same volume of ethyl acetate. The ethyl acetate solvent was evaporated, and the residues were dissolved in 1 mL methanol and then filter sterilised to obtain the crude extracts for both diffusion plate assay and UPLC‐MS assay. For the diffusion plate assay, 40 mL of MM agar with 40 μg/mL 5‐bromo‐4‐chloro‐3‐indolyl‐D‐galactopyranoside (X‐Gal) in a 130 × 130 mm Petri dish was cut into separate slices (1 cm in width). An aliquot of 1 μL crude extract of strain EC1 or mutant ∆*expR*_*Ecz*_ was spotted on to one end of an agar slice, and then the fresh cell culture of the AHL biosensor *A. tumefaciens* CF11 at an OD_600_ of 0.5 was spotted at progressively further distances from the crude extract. The plates were incubated a 28°C for 24 h. The blue spots of the AHL biosensor indicated the AHL activity. For further confirmation of OHHL production, a UPLC assay was performed on the Q Exactive Focus Hybrid Quadrupole‐Orbitrap mass spectrometer (Thermo Fisher Scientific) using an Acquity UPLC HSS‐T3 column (2.1 × 100 mm, 1.8 μm) and a solvent system consisting of methanol in water supplemented with 0.1% formic acid (0–6.5 min 2%, 6.5–10.5 min 100%, 10.5–12 min 2%). OHHL was detected in a positive mode showing a molecular ion (M + H)^+^ with an *m*/*z* of 214.11.

### 4.5 CWDE Assay

Cel and Peh activities were determined using previously established methods with Cel assay medium (0.1% carboxymethyl cellulose, 0.38% sodium phosphate, pH 7.0, and 0.8% agarose) and Peh assay medium (0.5% polygalacturonic acid, 0.2% sucrose, 0.2% ammonium sulphate and 1.5% agarose, pH 5.5), respectively (Lv et al. 2022). The assay plates were prepared by pouring about 30 mL assay medium into a 100 × 100 mm Petri dish. After solidification, 4.5‐mm diameter wells were made on the prepared assay plate. An aliquot of 20 μL of fresh cell culture of strain EC1 and its derivatives grown in LS5 medium with OD_600_ at 1.0 was added to each well in the assay plate. Cel assay plates incubated at 28°C for 16 h were stained with 0.1% Congo red (wt/vol) for 10 min and then decoloured with 1 M NaCl multiple times. Peh plates incubated at 28°C for 24 h were treated with 1 M HCl multiple times. Cel and Peh plates were photographed when the clear zone created by strain EC1 cells was visible, and Cel and Peh levels were quantified by measuring the diameter of the clear zone around the wells.

### 4.6 Zeamine Production Assay

Zeamine production of strain EC1 and derivatives was assayed using a method described previously (Liang et al. 2019). Briefly, the assayed plates were prepared by overlaying 25 mL solid LB agar with 7.5 mL 1% (wt/vol) agarose (with 5 × 10^8^ *E. coli* DH5 cells) in 10‐ by 10‐cm square plates. An aliquot of 30 μL of cell‐free supernatants (sterilised by a 0.22‐μm pore filter) was added to a 4.5‐mm diameter well punched in the plate. The plates were incubated at 37°C for 24 h, and the diameter of the inhibition zone around the well was measured. For semi‐quantification, one unit of zeamines was defined as the amount that can create a 2‐mm‐diameter inhibitory zone around the well. The number of zeamine units per millilitre was calculated by multiplying the units of zeamines measured from the bioassay by the fold change of the volume used in the test (30 μL) to 1 mL.

### 4.7 Swimming Motility Assay

Swimming motilities of *D. oryzae* strains were determined using a previously described method (Liang, Huang, et al. 2023). Bacterial cell cultures at an OD_600_ of 1.0 were spotted on the semisolid agar plates (per litre contains 10 g bacteriological peptone, 5 g NaCl, 3 g agar, pH 7.0). The diameter of chemotactic zone was measured after for an 18 h incubation at 28°C. IPTG at 100 μM or OHHL at 2 μM was supplied as a final concentration when necessary.

### 4.8 Biofilm Formation Assay

Biofilm formation assay was determined using a previously described method (Liang, Huang, et al. 2023). Bacterial cell cultures at an OD_600_ of 1.0 were inoculated (0.1% vol/vol) into 10‐cm glass tubes with YEB medium. The glass tubes were shaken at 28°C for 48 h. After incubation, cell cultures were removed from the glass tubes, and the biofilm mass on the glass tubes was washed twice with double‐distilled water and then stained with (0.1% wt/vol) crystal violet for 15 min. The unbound crystal violet was then removed, and the biofilm mass stained with crystal violet was rinsed three times with double‐distilled water and dried before photographing and quantification. For biofilm quantification, the crystal violet stained on the biofilm mass was dissolved in 75% ethanol and quantified by measuring the absorbance at 570 nm on a SYNERGY microplate reader (BioTek).

### 4.9 Virulence Against Chinese Cabbage Leaves and Potato Tubers

Determination of bacterial pathogenicity against Chinese cabbage leaves and potato tubers was performed following the methods described previously (Liang, Huang, et al. 2023). Briefly, Chinese cabbages and potatoes bought from the local shops were washed with double‐distilled water, dried with paper tissues, and then surface‐sterilised with 70% ethanol. Chinese cabbages and potato tubers were then cut to pieces about 8 × 8 cm or 5 mm in thickness, respectively, for inoculation. The sliced Chinese cabbages and potato tubers inoculated with bacterial suspensions in double‐distilled water (OD_600_ about 1.0) were incubated at 28°C for 36–48 h. OHHL was added at a final concentration of 2 μM before inoculation when necessary. The rotting areas caused by strain EC1 and its derivatives were photographed and quantified by ImageJ software (Schneider et al. 2012).

### 4.10 *Gfp* Transcriptional Fusion Assay

The reporter constructs p*P*_*expIEcz*_‐Gfp, p*P*_*expREcz*_‐Gfp, *F1*‐Gfp, *F2*‐Gfp, *F3*‐Gfp and *F4*‐Gfp were constructed using plasmid pPROBE‐NT (Miller et al. 2000), and transformed into strain EC1 and its derivatives for construction of reporter strains. The *Gfp* transcriptional fusion assay was performed following the method described previously (Liang et al. 2019). Briefly, a CytoFLEX flow cytometer (Beckman Coulter) was used for measuring the average fluorescence of 50,000 bacterial cells of each sample. The relative fluorescence of each reporter strain was expressed as the fluorescence of the bacterial strain normalised to the fluorescence of the corresponding control in each assay.

### 4.11 Sequence Alignment and Phylogenic Analysis

Sequence alignments were performed by Clustal X 2.1, and figures were generated by ESPript 3.0 (https://espript.ibcp.fr/ESPript/cgi‐bin/ESPript.cgi). Phylogenic analysis of ExpR_Ecz_ was performed in MEGA 6 (Tamura et al. 2013) by using the maximum‐likelihood method based on the best‐fit model (“LG + G”) (Le and Gascuel 2008) with 1000 bootstrap support. The amino acid sequences of ExpR_Ecz_ and its homologues were derived from UniProtKB database: ExpR_Ecz_ (A8D7L7), TraR (P33905), LuxR (P12746), LasR (P25084), EsaR (P54293) and ExpR_Ecc_ (Q47189). The *expR* 5′ noncoding regions of different *Dickeya* species were derived from NCBI database: *D. oryzae* EC1 (CP006929.1), *D. ananatis* A5410 (CP040816.1), *D. zeae* MS1 (CP053012.1), *D. fangzhongdai* DSM 101947 (CP025003.1), *D. solani* IPO 2222 (CP015137.1), *D. dianthicola* RNS04.9 (CP017638.1) and *D. dadantii* 3937(CP002038.1).

### 4.12 Statistical Analysis

All experiments were individually performed twice with at least three replicates each time. Statistical comparison was performed by using two‐tailed unpaired Student's *t*‐test or Mann–Whitney test in GraphPad Prism 7.0 software (GraphPad).

## Author Contributions

**Ling He:** investigation, validation. **Lian‐Hui Zhang:** conceptualization, writing – review and editing, supervision, funding acquisition, project administration. **Qunyi Chen:** methodology, validation, investigation. **Zhibin Liang:** conceptualization, writing – original draft, writing – review and editing, investigation, methodology, validation. **Weihan Gu:** methodology, validation, investigation. **Xinbo Wang:** methodology, validation, investigation. **Zhongqiao Chen:** methodology, validation, investigation. **Huidi Liu:** methodology, investigation, validation. **Xiaoyan Wu:** methodology, validation, investigation.

## Funding

This work was supported by National Natural Science Foundation of China (U22A20480).

## Conflicts of Interest

The authors declare no conflicts of interest.

## Supporting information


**Figure S1:** Bioinformatics analysis of ExpREcz and its homologues. The amino acid sequences of ExpREcz and its homologues were derived from UniProtKB database: ExpREcz (A8D7L7), TraR (P33905), LuxR (P12746), LasR (P25084), EsaR (P54293) and ExpREcc (Q47189). (A) Sequence alignment of ExpREcz and its homologues. The number in the parenthesis indicates the amino acid sequence similarity between ExpREcz and each of its homologue. The asterisks indicate two conserved amino acid residues (Y50 and W54 of ExpREcz) required for OHHL interaction. (B) Phylogenic relationship of ExpREcz and its homologues. The amino acid of ExpREcz and its homologues were aligned by ClustalW. The phylogenic tress was constructed using MEGA6 (Tamura et al. 2013) with Maximum Likelihood method based on the best‐fit model (“LG + G”) (Le and Gascuel 2008). Bootstrap values higher than 50% are shown.

**Figure S2:** AHL production of EC1 and its derivatives. *Agrobacterium tumefaciens* CF11 was used as the AHL biosensor, and the amount of AHL signal of cell cultures (A) or crude extracts (B, Extract) were determined.

**Figure S3:** Cel and Peh production of EC1 and its derivatives. Cel (A) and Peh (B) production of EC1 and its derivatives were compared in the Cel and Peh plates with carboxymethyl cellulose sodium and polygalacturonic acid, respectively.

**Figure S4:** Swimming motility and biofilm formation of EC1 and its derivatives. (A) Swimming motility of EC1 and its derivatives were assayed in the semisolid agar plates and photographed. (B) Biofilm formation of EC1 and its derivatives measured in the glass tubes and photographed after crystal violet staining.

**Figure S5:** OHHL restores the defect of swimming motility and the virulence of *expIEcz* mutant. (A) Swimming motility of strain EC1 and the *expIEcz* mutant Δ*expIEcz* with or without exogenous addition OHHL (2 μM). Swimming motility was assayed in the semisolid agar plates and the diameter of chemotactic zones were measured. Data are presented as mean ± SD, *n* = 3. (B) and (C) Virulence of strain EC1 and its derivatives against Chinese cabbages and potato tubers. EC1 and the *expIEcz* mutant Δ*expIEcz* with or without exogenous addition OHHL (2 μM) were inoculated into the sliced Chinese cabbages (B) or potato tubers (C). The rotting areas were measured at 48 h post inoculation. In (A), data are presented as mean ± SD, *n* = 3. Statistical analysis was performed using a two‐tailed unpaired Student's *t*‐test. Symbol: ***, *p* < 0.001. In (B) and (C), data are presented as mean ± SD, *n* = 4. Statistical analysis was performed using Mann–Whitney test. Symbol: *, *p* < 0.05.

**Figure S6:** DNA sequence of *PexpIEcz*. The −35 and −10 elements, SD sequence predicted in our previous study (Hussain et al. 2008), and a potential ArcA binding site detected in this study by the website BPROM (http://www.softberry.com/berry.phtml?topic=bprom&group=programs&subgroup=gfindb) were indicated by using blue, red, green and purple frames, respectively. The start codon of *expRIcz* (ATG) is also presented using orange font.

**Figure S7:** Sequence alignment of the 5′ noncoding region of *expR* among different *Dickeya* strains. Sequence alignment of the 5′ noncoding region of *expR* in *Dickeya oryzae* EC1 (NCBI accession no. CP006929.1), *Dickeya ananatis* A5410 (NCBI accession no. CP040816.1), *Dickeya zeae* MS1 (NCBI accession no. CP053012.1), *Dickeya fangzhongdai* DSM 101947 (NCBI accession no. CP025003.1), *Dickeya solani* IPO 2222 (NCBI accession no. CP015137.1), *Dickeya dianthicola* RNS04.9 (NCBI accession no. CP017638.1) and *Dickeya dadantii* 3937 (NCBI accession no. CP002038.1) were performed by Clustal X 2.1. The EDRR (ExpREcz‐dependent regulatory region) in different *Dickeya* strains are indicated by the black frames.

**Table S1:** Bacterial strains and plasmids used in this study.

**Table S2:** Primers used in this study.

## Data Availability

The data that support the findings of this study are available from the corresponding author upon reasonable request.
