# Supplementary material for: Functional Characterisation of the Quorum‐Sensing Regulator ExpREcz in Modulation of Dickeya oryzae Motility and Virulence
Source: Mol Plant Pathol. 2026 Jun 22;27(6):e70274. doi: 10.1111/mpp.70274 (PMC13286868; doi:10.1111/mpp.70274)
Supplement: Supplementary file 1 — Figure S1: Bioinformatics analysis of ExpREcz and its homologues. The amino acid sequences of ExpREcz and its homologues were derived from UniProtKB database: ExpREcz (A8D7L7), TraR (P33905), LuxR (P12746), LasR (P25084), EsaR (P54293) and ExpREcc (Q47189). (A) Sequence alignment of ExpREcz and its homologues. The number in the parenthesis indicates the amino acid sequence similarity between ExpREcz and each of its homologue. The asterisks indicate two conserved amino acid residues (Y50 and W54 of ExpREcz) required for OHHL interaction. (B) Phylogenic relationship of ExpREcz and its homologues. The amino acid of ExpREcz and its homologues were aligned by ClustalW. The phylogenic tress was constructed using MEGA6 (Tamura et al. 2013) with Maximum Likelihood method based on the best‐fit model (“LG + G”) (Le and Gascuel 2008). Bootstrap values higher than 50% are shown. [file MPP-27-e70274-s006.pdf]

A

```
ExpR_Ecz        1   ........MSVSFSNVDFINSTIQN..YLNRKLKSYGDLKYAYLIMNKKKP..TDVVIIS
TraR     (48)   1   ........MQHWLDKLTDLAAIEGDECILKTGLADIAD.HFGFTGYAYLHIQHRHITAVT
LasR     (43)   1   ......MALVDGFLELERSSGKLEWSAILQKMASDLGFSKILFGLLPKDSQDYENAFIVG
LuxR     (48)   1   MKNINADDTYRIINKIKACRSNNDINQCLSDMTKMVHCEYYLLAIIYPHSMVKSDISILD
EsaR     (72)   1   ........MFSFFLENQTITDTLQT..YIQRKLSPLGSPDYAYTVVSKKNP..SNVLIIS
ExpR_Ecc (81)   1   ........MSQLFYNNETISRIIKS..QFDMALSHYGDIKYAYMVLNKKKP..TEILIIS

ExpR_Ecz       49   NYPSEWVEIYRSNNYQHIDPVILTAINKISPFSWDDNLVISSKLK.FSRIFNLSKEYDIV
TraR     (48)  52   NYHRQWQSTYFDKKFEALDPVVKRARSRKHIFTWSGEHERPTLSKDERAFYDHASDFGIR
LasR     (43)  55   NYPAAWREHYDRAGYARVDPTVSHCTQSVLPIFWEP..SIYQTRK.QHEFFEEASAAGLV
LuxR     (48)  61   NYPKKWRQYYDDANLIKYDPIVDYSNSNHSPINWNIFENNAVNKK.SPNVIKEAKTSGLI
EsaR     (72)  49   SYPDEWIRLYRANNFQLTDPVILTAFKRTSPFAWDENITLMSDLR.FTKIFSLSKQYNIV
ExpR_Ecc (81)  49   NHHDEWREIYQANNYQHIDPVVIAALNKITPFPWDEDLLVSTQLK.MSKIFNLSREHNIT

ExpR_Ecz      108   NGYTFVLHDPGNNLATLSFMFEENRSGELEEIVHNNKEKLQMLLISAHEKLTSLY.REMS
TraR     (48) 112   SGITIPIKTANGFMSMFTMASD.KPVIDLDREIDAVAAAATIGQIHARISFLRTT.....
LasR     (43) 112   YGLTMPLHGARGELGALSLSVEAENRAEANRFMESVLPTLWMLKDYALQSGAGLA.....
LuxR     (48) 120   TGFSFPIHTANNGFGMLSFAHSEKDNYIDSLFLHACMN.IPLIVPSLVDNYRKIN.....
EsaR     (72) 108   NGFTYVLHDHMNNLALLSVIIKGNDQTALEQRLAAEQGTMQMLLIDFNEQMYRLAGTEGE
ExpR_Ecc (81) 108   NGYTFVLHDHSNNLVMLSIMIDESNVSNIDDVIESNKDKLQMTLMTIHAETISLY.REMI

ExpR_Ecz      167   KNKNNSKSQEPNLFSQRENEILYWASMGKTYQEIALILGITTSTVKFHIGNVVKKLGVLN
TraR     (48) 166   .....PTAEDAAWLDPKEATYLRWIAVGKTMEEIADVEGVKYNSVRVKLREAMKRFDVRS
LasR     (43) 167   ...FEHPVSKPVVLTSREKEVLQWCAIGKTSWEISVICNCSEANVNFHMGNIRRKFGVTS
LuxR     (48) 174   ....IANNKSNNDLTKREKECLAWACEGKSSWDISKILGCSERTVTFHLTNAQMKLNTTN
EsaR     (72) 168   RAPALNQSADKTIFSSRENEVLYWASMGKTYAEIAAITGISVSTVKFHIKNVVVKLGVSN
ExpR_Ecc (81) 167   RNKEDERSNDKDIFSQRENEILYWASMGKTYQEIALILDIKTGTVKFHIGNVVKKLGVLN

ExpR_Ecz      227   AKHAIRLGVEMNLIKPVEPVKARS
TraR     (48) 221   KAHLTALAIRRKLI..........
LasR     (43) 224   RRVAAIMAVNLGLITL........
LuxR     (48) 230   RCQSISKAILTGAIDCPYFKN...
EsaR     (72) 228   ARQAIRLGVELDLIRPAASAAR..
ExpR_Ecc (81) 227   AKHAIRLGIELQLIRPVQS.....
```

B

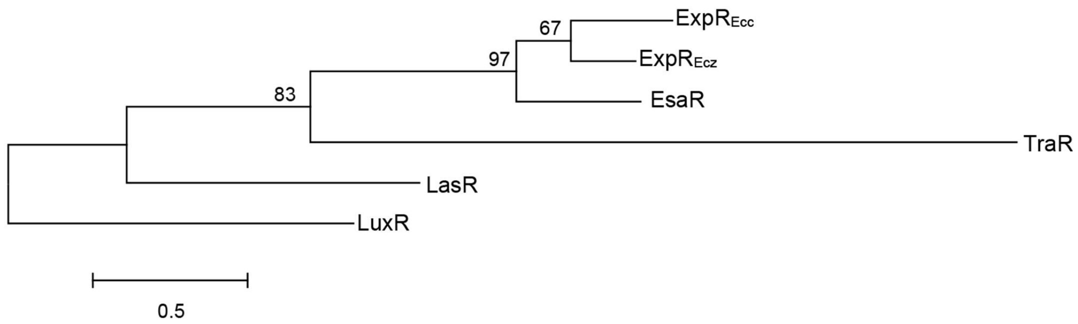

Figure S1 Bioinformatics analysis of $ExpR_{Ecz}$ and its homologs. The amino acid sequences of $ExpR_{Ecz}$ and its homologs were derived from UniProtKB database: $ExpR_{Ecz}$ (A8D7L7), TraR (P33905), LuxR (P12746), LasR (P25084), EsaR (P54293), and $ExpR_{Ecc}$ (Q47189). (A) Sequence alignment of $ExpR_{Ecz}$ and its homologs. The number in the parenthesis indicates the amino acid sequence similarity between $ExpR_{Ecz}$ and each of its homolog. The asterisks indicate two conserved amino acid residues (Y50 and W54 of $ExpR_{Ecz}$) required for OHHL interaction. (B) Phylogenic relationship of $ExpR_{Ecz}$ and its homologs. The amino acid of $ExpR_{Ecz}$ and its homologs were aligned by ClustalW. The phylogenic tress was constructed using MEGA6 (Tamura et al. 2013) with Maximum Likelihood method based on the best-fit model ("LG + G") (Le et al. 2008). Bootstrap values higher than 50% are shown.
