# Supplementary material for: Functional Characterisation of the Quorum‐Sensing Regulator ExpREcz in Modulation of Dickeya oryzae Motility and Virulence
Source: Mol Plant Pathol. 2026 Jun 22;27(6):e70274. doi: 10.1111/mpp.70274 (PMC13286868; doi:10.1111/mpp.70274)
Supplement: Supplementary file 2 — Figure S2: AHL production of EC1 and its derivatives. Agrobacterium tumefaciens CF11 was used as the AHL biosensor, and the amount of AHL signal of cell cultures (A) or crude extracts (B, Extract) were determined. [file MPP-27-e70274-s002.pdf]

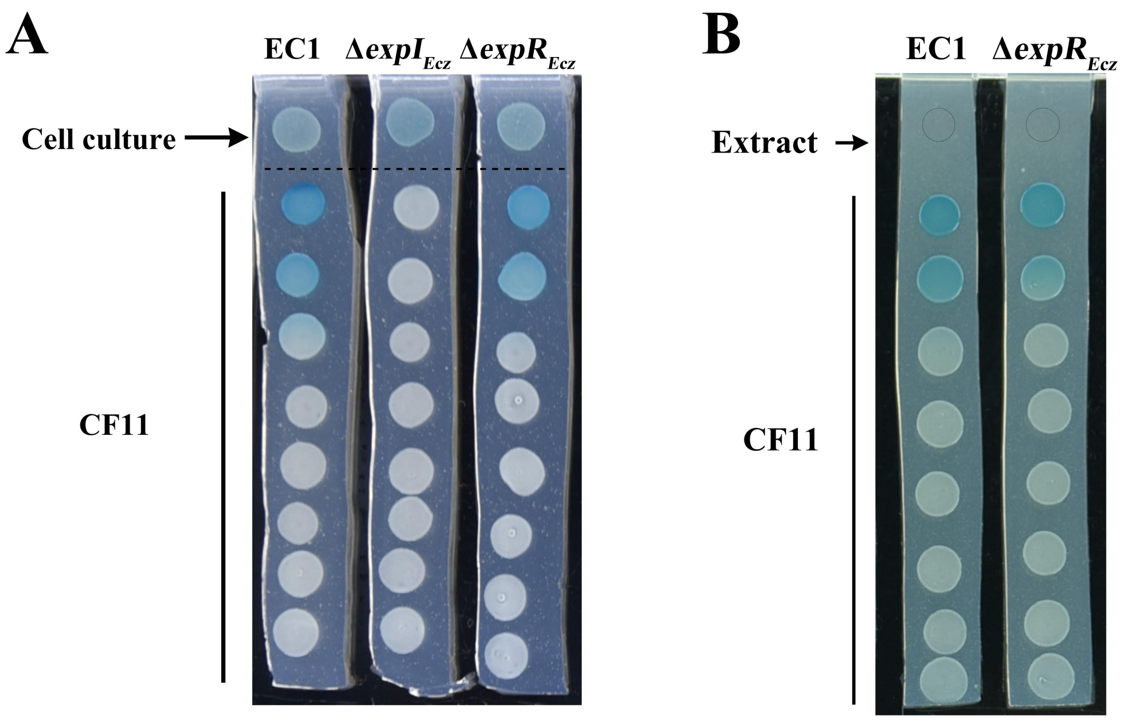

Figure S2 AHL production of EC1 and its derivatives. *Agrobacterium tumefaciens* CF11 was used as the AHL biosensor, and the amount of AHL signal of cell cultures (A) or crude extracts (B, Extract) were determined.
