# Supplementary material for: Functional Characterisation of the Quorum‐Sensing Regulator ExpREcz in Modulation of Dickeya oryzae Motility and Virulence
Source: Mol Plant Pathol. 2026 Jun 22;27(6):e70274. doi: 10.1111/mpp.70274 (PMC13286868; doi:10.1111/mpp.70274)
Supplement: Supplementary file 3 — Figure S3: Cel and Peh production of EC1 and its derivatives. Cel (A) and Peh (B) production of EC1 and its derivatives were compared in the Cel and Peh plates with carboxymethyl cellulose sodium and polygalacturonic acid, respectively. [file MPP-27-e70274-s004.pdf]

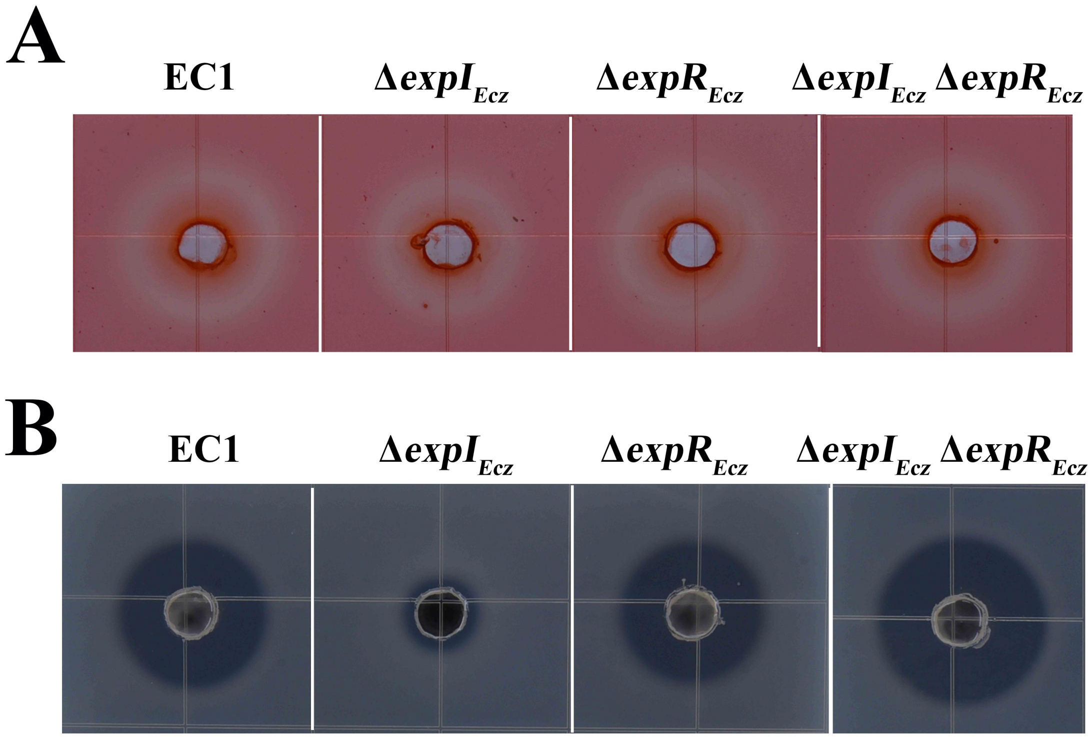

Figure S3 Cel and Peh production of EC1 and its derivatives. Cel (A) and Peh (B) production of EC1 and its derivatives were compared in the Cel and Peh plates with carboxymethyl cellulose sodium and polygalacturonic acid, respectively.
