# Supplementary material for: Functional Characterisation of the Quorum‐Sensing Regulator ExpREcz in Modulation of Dickeya oryzae Motility and Virulence
Source: Mol Plant Pathol. 2026 Jun 22;27(6):e70274. doi: 10.1111/mpp.70274 (PMC13286868; doi:10.1111/mpp.70274)
Supplement: Supplementary file 4 — Figure S4: Swimming motility and biofilm formation of EC1 and its derivatives. (A) Swimming motility of EC1 and its derivatives were assayed in the semisolid agar plates and photographed. (B) Biofilm formation of EC1 and its derivatives measured in the glass tubes and photographed after crystal violet staining. [file MPP-27-e70274-s003.pdf]

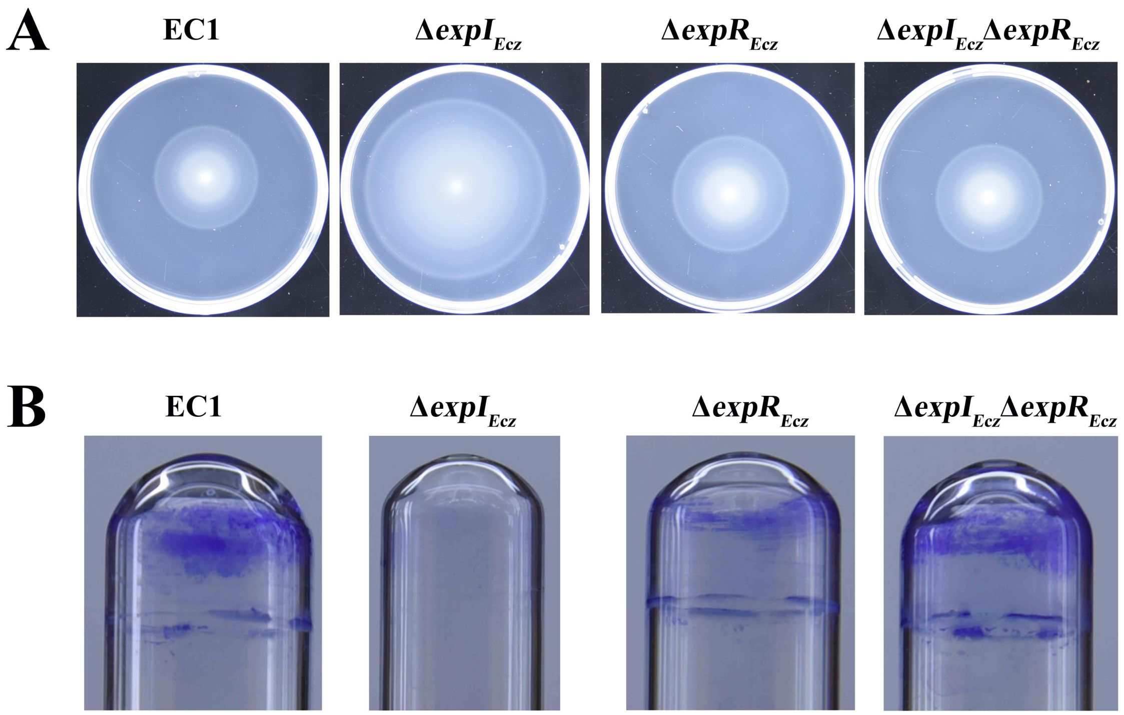

Figure S4 Swimming motility and biofilm formation of EC1 and its derivatives. (A) Swimming motility of EC1 and its derivatives were assayed in the semisolid agar plates and photographed. (B) Biofilm formation of EC1 and its derivatives measured in the glass tubes and photographed after crystal violet staining.
