# Supplementary material for: Functional Characterisation of the Quorum‐Sensing Regulator ExpREcz in Modulation of Dickeya oryzae Motility and Virulence
Source: Mol Plant Pathol. 2026 Jun 22;27(6):e70274. doi: 10.1111/mpp.70274 (PMC13286868; doi:10.1111/mpp.70274)
Supplement: Supplementary file 5 — Figure S5: OHHL restores the defect of swimming motility and the virulence of expIEcz mutant. (A) Swimming motility of strain EC1 and the expIEcz mutant ΔexpIEcz with or without exogenous addition OHHL (2 μM). Swimming motility was assayed in the semisolid agar plates and the diameter of chemotactic zones were measured. Data are presented as mean ± SD, n = 3. (B) and (C) Virulence of strain EC1 and its derivatives against Chinese cabbages and potato tubers. EC1 and the expIEcz mutant ΔexpIEcz with or without exogenous addition OHHL (2 μM) were inoculated into the sliced Chinese cabbages (B) or potato tubers (C). The rotting areas were measured at 48 h post inoculation. In (A), data are presented as mean ± SD, n = 3. Statistical analysis was performed using a two‐tailed unpaired Student's t‐test. Symbol: ***, p < 0.001. In (B) and (C), data are presented as mean ± SD, n = 4. Statistical analysis was performed using Mann–Whitney test. Symbol: *, p < 0.05. [file MPP-27-e70274-s007.pdf]

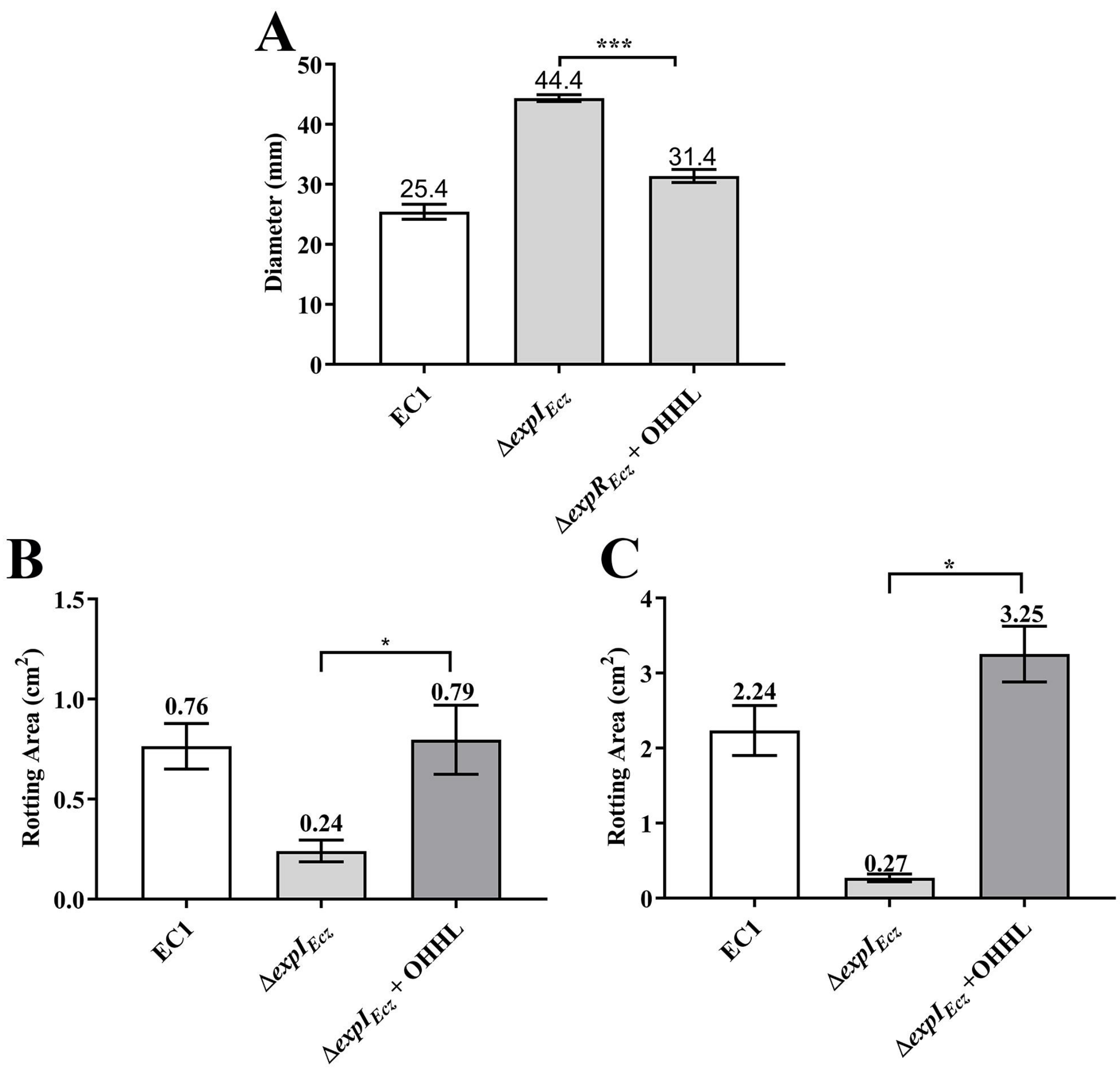

Figure S5 OHHL restores the defect of swimming motility and the virulence of *expI*$_{Ecz}$ mutant. (A) Swimming motility of strain EC1 and the *expI*$_{Ecz}$ mutant Δ*expI*$_{Ecz}$ with or without exogenous addition OHHL (2 μM). Swimming motility was assayed in the semisolid agar plates and the diameter of chemotactic zones were measured. Data are presented as mean ± SD, n = 3. (B) and (C) Virulence of strain EC1 and its derivatives against Chinese cabbages and potato tubers. EC1 and the *expI*$_{Ecz}$ mutant Δ*expI*$_{Ecz}$ with or without exogenous addition OHHL (2 μM) were inoculated into the sliced Chinese cabbages (B) or potato tubers (C). The rotting areas were measured at 48 h post inoculation. In (A), data are presented as mean ± SD, n = 3. Statistical analysis was performed using a two-tailed unpaired Student's t test. Symbol: ***, $P < 0.001$. In (B) and (C), data are presented as mean ± SD, n = 4. Statistical analysis was performed using Mann-Whitney test. Symbol: *, $P < 0.05$.
