# Supplementary material for: Functional Characterisation of the Quorum‐Sensing Regulator ExpREcz in Modulation of Dickeya oryzae Motility and Virulence
Source: Mol Plant Pathol. 2026 Jun 22;27(6):e70274. doi: 10.1111/mpp.70274 (PMC13286868; doi:10.1111/mpp.70274)
Supplement: Supplementary file 6 — Figure S6: DNA sequence of PexpIEcz. The −35 and −10 elements, SD sequence predicted in our previous study (Hussain et al. 2008), and a potential ArcA binding site detected in this study by the website BPROM (http://www.softberry.com/berry.phtml?topic=bprom&group=programs&subgroup=gfindb) were indicated by using blue, red, green and purple frames, respectively. The start codon of expRIcz (ATG) is also presented using orange font. [file MPP-27-e70274-s008.pdf]

CATACGTTGTTAAGTACGCCTGCATATTATTCCAAATATTAATTATTCTTTCGGTG

GGTATTATCCCCTGACTAACCGGGAGTTATCTCGCTTCTGGCATACGCCGTGAA

CGTGGATAAGGAAACTATATGTCAACTAATTAAAAAAAGTTTCCCTTACATGAC

-35 element

CTCAAATAATCACGCTGGAAAATCAAGCAATAAAAACGTTAAAATACCCTCAC

-10 element **ArcA binding site**

CAGGTGAGCTATTGCGCAAAAAGACATGAAGTTAAGGATAGGGGGAAATATG

SD sequence

Figure S6 DNA sequence of $P_{expIEcz}$. The -35 and -10 elements, SD sequence predicted in our previous study (Hussain et al., 2008), and a potential ArcA binding site detected in this study by the website BPROM (http://www.softberry.com/berry.phtml?topic=bprom&group=programs&subgroup=gfindb) were indicated by using blue, red, green, and purple frames, respectively. The start codon of *expR*$_{Icz}$ (ATG) is also presented using orange font.
