# Supplementary material for: Functional Characterisation of the Quorum‐Sensing Regulator ExpREcz in Modulation of Dickeya oryzae Motility and Virulence
Source: Mol Plant Pathol. 2026 Jun 22;27(6):e70274. doi: 10.1111/mpp.70274 (PMC13286868; doi:10.1111/mpp.70274)
Supplement: Supplementary file 7 — Figure S7: Sequence alignment of the 5′ noncoding region of expR among different Dickeya strains. Sequence alignment of the 5′ noncoding region of expR in Dickeya oryzae EC1 (NCBI accession no. CP006929.1), Dickeya ananatis A5410 (NCBI accession no. CP040816.1), Dickeya zeae MS1 (NCBI accession no. CP053012.1), Dickeya fangzhongdai DSM 101947 (NCBI accession no. CP025003.1), Dickeya solani IPO 2222 (NCBI accession no. CP015137.1), Dickeya dianthicola RNS04.9 (NCBI accession no. CP017638.1) and Dickeya dadantii 3937 (NCBI accession no. CP002038.1) were performed by Clustal X 2.1. The EDRR (ExpREcz‐dependent regulatory region) in different Dickeya strains are indicated by the black frames. [file MPP-27-e70274-s009.pdf]

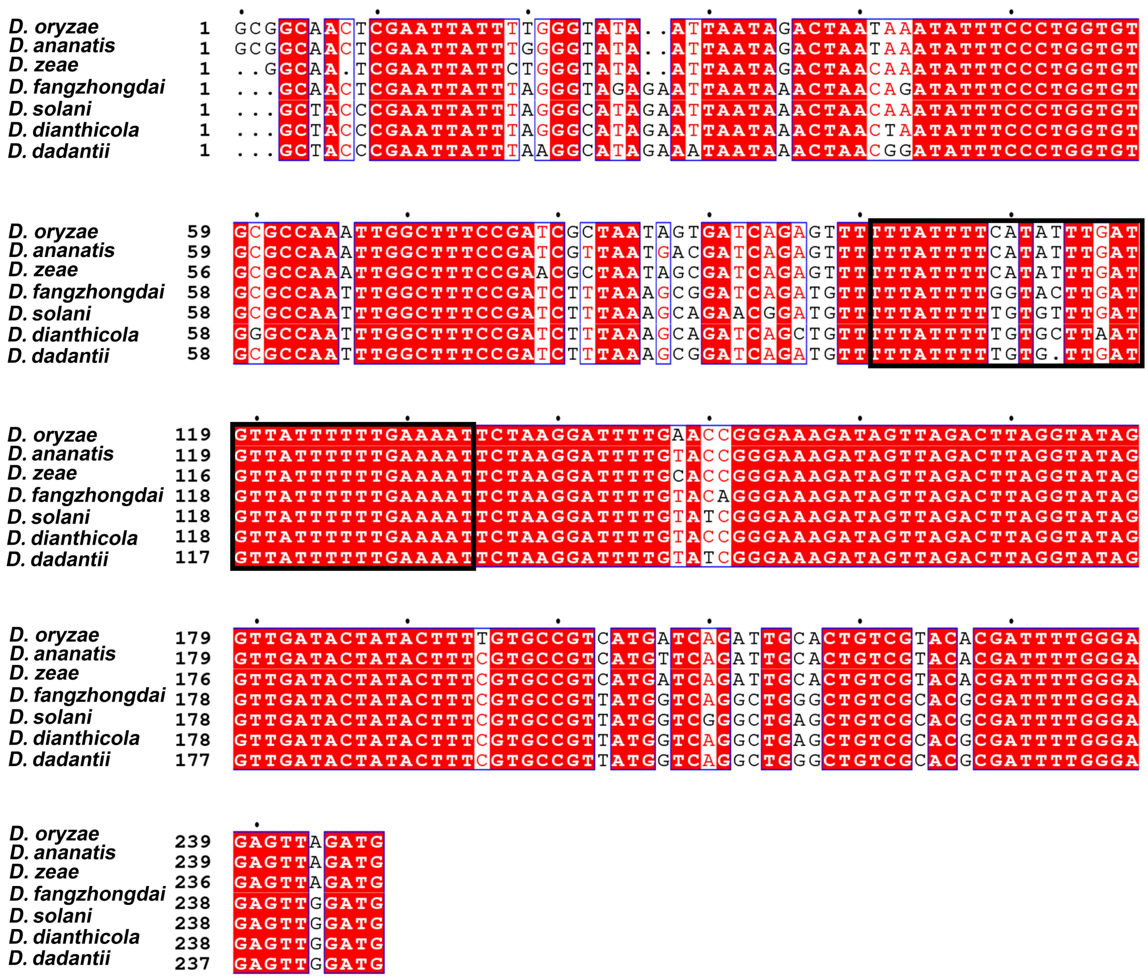

Figure S7 Sequence alignment of the 5'noncoding region of *expR* among different *Dickeya* strains. Sequence alignment of the 5'noncoding region of *expR* in *Dickeya oryzae* EC1 (NCBI accession no. CP006929.1), *Dickeya ananatis* A5410 (NCBI accession no. CP040816.1), *Dickeya zeae* MS1 (NCBI accession no. CP053012 .1), *Dickeya fangzhongdai* DSM 101947 (NCBI accession no. CP025003.1), *Dickeya solani* IPO 2222 (NCBI accession no. CP015137.1), *Dickeya dianthicola* RNS04.9 (NCBI accession no. CP017638.1), and *Dickeya dadantii* 3937 (NCBI accession no. CP002038.1) were performed by Clustal X 2.1. The EDRR (ExpR$_{Ecz}$-dependent regulatory region) in different *Dickeya* strains are indicated by the black frames.
