# Supplementary material for: Functional Characterisation of the Quorum‐Sensing Regulator ExpREcz in Modulation of Dickeya oryzae Motility and Virulence
Source: Mol Plant Pathol. 2026 Jun 22;27(6):e70274. doi: 10.1111/mpp.70274 (PMC13286868; doi:10.1111/mpp.70274)
Supplement: Supplementary file 8 — Table S1: Bacterial strains and plasmids used in this study. [file MPP-27-e70274-s001.pdf]

TableS1 Bacterial strains and plasmids used in this study

Strains or plasmids	Relevant characteristics[a]	Source or reference
Dickeya oryzae		
EC1	Wild-type strain of *D. oryzae*	(Hussain et al., 2008)
$\Delta expI_{Ecz}$	In-frame deletion of $expR_{Ecz}$ in EC1	This study
$\Delta expR_{Ecz}$	In-frame deletion of $expR_{Ecz}$ in EC1	This study
$\Delta expI_{Ecz}\Delta expR_{Ecz}$	In-frame deletion of both $expI_{Ecz}$ and $expR_{Ecz}$ in EC1	This study
EC1(p$P_{expREcz}$-Gfp)	EC1 with the p$P_{expREcz}$-Gfp vector, Kan^r	This study
$\Delta expI_{Ecz}$(p$P_{expREcz}$-Gfp)	$\Delta expI_{Ecz}$ with the p$P_{expREcz}$-Gfp vector, Kan^r	This study
$\Delta expR_{Ecz}$(p$P_{expREcz}$-Gfp)	$\Delta expR_{Ecz}$ with the p$P_{expREcz}$-Gfp vector, Kan^r	This study
$\Delta expI_{Ecz}\Delta expR_{Ecz}$(p$P_{expREcz}$-Gfp)	$\Delta expI_{Ecz}\Delta expR_{Ecz}$ with the p$P_{expREcz}$-Gfp vector, Kan^r	This study
EC1(p$P_{expIEcz}$-Gfp)	EC1 with the p$P_{expIEcz}$-Gfp vector, Kan^r	This study
$\Delta expI_{Ecz}$(p$P_{expIEcz}$-Gfp)	$\Delta expI_{Ecz}$ with the p$P_{expIEcz}$-Gfp vector, Kan^r	This study
$\Delta expR_{Ecz}$(p$P_{expIEcz}$-Gfp)	$\Delta expR_{Ecz}$ with the p$P_{expIEcz}$-Gfp vector, Kan^r	This study
$\Delta expI_{Ecz}$(*F1*-Gfp)	$\Delta expI_{Ecz}$ with the *F1*-Gfp vector, Kan^r	This study
$\Delta expI_{Ecz}$(*F2*-Gfp)	$\Delta expI_{Ecz}$ with the *F2*-Gfp vector, Kan^r	This study
$\Delta expI_{Ecz}$(*F3*-Gfp)	$\Delta expI_{Ecz}$ with the *F3*-Gfp vector, Kan^r	This study
$\Delta expI_{Ecz}$(*F4*-Gfp)	$\Delta expI_{Ecz}$ with the *F4*-Gfp vector, Kan^r	This study
$\Delta expI_{Ecz}\Delta expR_{Ecz}$(pET28a)	$\Delta expI_{Ecz}\Delta expR_{Ecz}$ with the pET28a vector, Kan^r	This study
$\Delta expI_{Ecz}\Delta expR_{Ecz}$(pET28a-$expR_{Ecz}$)	$\Delta expI_{Ecz}\Delta expR_{Ecz}$ with the pET28a-$expR_{Ecz}$ vector, Kan^r	This study
$\Delta expI_{Ecz}\Delta expR_{Ecz}$(pET28a-$expR_{Ecz}^{Y50A}$)	$\Delta expI_{Ecz}\Delta expR_{Ecz}$ with the pET28a-$expR_{Ecz}^{Y50A}$ vector, Kan^r	This study
$\Delta expI_{Ecz}\Delta expR_{Ecz}$(pET28a-$expR_{Ecz}^{W54A}$)	$\Delta expI_{Ecz}\Delta expR_{Ecz}$ with the pET28a-$expR_{Ecz}^{W54A}$ vector, Kan^r	This study
Agrobacterium tumefaciens CF11		
CF11	AHL biosensor, with two plasmids expressing TraR and the reporter gene *lacZ* under the control of *tra* promoter, respectively	Laboratory collection
Escherichia coli		
DH5α	F-, φ80*lac*ZΔM15, Δ(*lac*ZYA-*arg*F)U169, *end*A1, *rec*A1, *hsd*R17 (r_k- ,m_k+), *sup*E44, λ-, *thi*-1, *gyr*A96, *rel*A1, *pho*A	TransGen Biotech, China
CC118	Host strain for the replication of pKNG101 and derivative plasmids	Laboratory collection
HB101 (pRK2013)	*Thr leu thi recA hsdR hsdM pro*, Kan^r	Laboratory collection
Plasmids		

pKNG101	Suicide vector for gene in-frame deletion, Str^r	Laboratory collection
pKNG-*expI*$_{Ecz}$	pKNG101 harboring the flanking region of *expI*$_{Ecz}$, Str^r	This study
pKNG-*expR*$_{Ecz}$	pKNG101 harboring the flanking region of *expR*$_{Ecz}$, Str^r	This study
pPROBE-NT	Promoterless *gfp* transcriptional reporter plasmid, Kan^r	Laboratory collection
p$P_{expIEcz}$-Gfp	*Gfp* transcriptional fusion with the promoter region of *expI*$_{Ecz}$, Kan^r	This study
p$P_{expREcz}$-Gfp	*Gfp* transcriptional fusion with the promoter region of *expR*$_{Ecz}$, Kan^r	This study
F1-Gfp	*Gfp* transcriptional fusion with an 164-bp promoter region of *expR*$_{Ecz}$, Kan^r	This study
F2-Gfp	*Gfp* transcriptional fusion with an 145-bp promoter region of *expR*$_{Ecz}$, Kan^r	This study
F3-Gfp	*Gfp* transcriptional fusion with an 111-bp promoter region of *expR*$_{Ecz}$, Kan^r	This study
F4-Gfp	*Gfp* transcriptional fusion with a 70-bp promoter region of *expR*$_{Ecz}$, Kan^r	This study
pET28a	expression vector with T7 promoter and *lac* operator, Kan^r	Laboratory collection
pET28a-*expR*$_{Ecz}$	pET28a carrying the coding sequence of *expR*$_{Ecz}$, whose expression is under the control of *lac* operator, Kan^r	This study
pET28a-*expR*$_{Ecz}^{Y50A}$	pET28a carrying the coding sequence of *expR*$_{Ecz}$ with a single alanine alteration in Y50, whose expression is under the control of *lac* operator, Kan^r	This study
pET28a-*expR*$_{Ecz}^{W54A}$	pET28a carrying the coding sequence of *expR*$_{Ecz}$ with a single alanine alteration in W54, whose expression is under the control of *lac* operator, Kan^r	This study

[a]Abbreviations: Kan^r, kanamycin resistance; Str^r, streptomycin resistance.

Hussain, M. B., Zhang, H. B., Xu, J. L., Liu, Q., Jiang, Z. and Zhang, L. H. (2008) The acyl-homoserine lactone-type quorum-sensing system modulates cell motility and virulence of *Erwinia chrysanthemi* pv. *zeae*. *J Bacteriol,* **190,** 1045-1053.
