# Supplementary material for: Functional Characterisation of the Quorum‐Sensing Regulator ExpREcz in Modulation of Dickeya oryzae Motility and Virulence
Source: Mol Plant Pathol. 2026 Jun 22;27(6):e70274. doi: 10.1111/mpp.70274 (PMC13286868; doi:10.1111/mpp.70274)
Supplement: Supplementary file 9 — Table S2: Primers used in this study. [file MPP-27-e70274-s005.pdf]

Table S2 Primers used in this study

Name	Sequence (5'–3')[a]	Description
Primers for gene in-frame deletion		
$expI_{Ecz}$-1	gtcgacggatccccgggtacCCCCCTTCTCCTGGGATGATAATC	
$expI_{Ecz}$-2	GTCAAATAACAAGCTGGATGAGGTGGAGCAGCTTAAGACCTTGCCT	$expI_{Ecz}$ in-frame deletion
$expI_{Ecz}$-3	AGGCAAGGTCTTAAGCTGCTCCACCTCATCCAGCTTGTTATTTGAC	
$expI_{Ecz}$-4	cttatggtacccggggatccACCCAACTTCACAAAAAGTGCGAT	
$expR_{Ecz}$-1	gtcgacggatccccgggtacATTTCGCTGCGTTTACAGGTGATT	
$expR_{Ecz}$-2	ACTGGTTCCACTGGTTTGATCAGCATCTAACTCTCCCAAAATCGTGT	$expR_{Ecz}$ in-frame deletion
$expR_{Ecz}$-3	ACACGATTTTGGGAGAGTTAGATGCTGATCAAACCAGTGGAACCAGT	
$expR_{Ecz}$-4	cttatggtacccggggatccATGGGATGGAGTTCGACGAGT	
$expIR_{Ecz}$-1	gtcgacggatccccgggtacATTTCGCTGCGTTTACAGGTGATT	
$expIR_{Ecz}$-2	GTCAAATAACAAGCTGGATGAGGTGCATCTAACTCTCCCAAAATCGTGT	double deletion of $expI_{Ecz}$ and $expR_{Ecz}$
$expIR_{Ecz}$-3	ACACGATTTTGGGAGAGTTAGATGCACCTCATCCAGCTTGTTATTTGAC	
$expIR_{Ecz}$-4	cttatggtacccggggatccCCCCCTTCTCCTGGGATGATAATC	
Primers for amplifying the promoter region of $expI_{Ecz}$ and $expR_{Ecz}$ for constructing reporter strains		
P-$expI_{Ecz}$-F	ggaattggggatcggaagcttCATACGTTGTTAAGTACGCCTG	construction of p$P_{expIEcz}$-Gfp
P-$expI_{Ecz}$-R	cggtacccggggatccTATTTCTAACATATTTCCCCCTATC	
P-$expR_{Ecz}$-R	cggtacccggggatccAGAGAATGATACAGACATCTAACTC	construction of p$P_{expREcz}$-Gfp, *F1*-Gfp, *F2*-Gfp, *F3*-Gfp, and *F4*-Gfp
P-$expR_{Ecz}$-F	ggaattggggatcggaagcttGCGGCAACTCGAATTATTTTGGG	construction of p$P_{expREcz}$-Gfp
P-*F1(164)*-F	ggaattggggatcggaagcttCTAATAGTGATCAGAGTTTTTTATT	construction of *F1*-Gfp
P-*F2(145)*-F	ggaattggggatcggaagcttTTTATTTTCATATTTGATGTTATTT	construction of *F2*-Gfp
P-*F3(111)*-F	ggaattggggatcggaagcttTCTAAGGATTTTGAACCGGGAAAGA	construction of *F3*-Gfp
P-*F4(70)*-F	ggaattggggatcggaagcttTAGGTTGATACTATACTTTTGTGCC	construction of *F4*-Gfp
Primers for constructing the plasmids for the induced expression of $expR_{Ecz}$, $expR_{Ecz}^{Y50A}$, and $expR_{Ecz}^{W50A}$		
pET28a-1	gtgccgcgcggcagcGATGTCTGTATCATTCTCTAACGTT	induced expression of $expR_{Ecz}$, $expR_{Ecz}^{Y50A}$, and $expR_{Ecz}^{W54A}$
pET28a-4	cggagctcgaattcggatccGCTTAAGACCTTGCCTTTACTGG	
pET28a-Y50A-2	CCCATTCCGATGGAGCATTAGAAATAATGACCACATCCGTCGGC	induced expression of $expR_{Ecz}^{Y50A}$
pET28a-Y50A-3	GCCGACGGATGTGGTCATTATTTCTAATGCTCCATCGGAATGGG	
pET28a-W54A-2	AAATCTCAACCGCTTCCGATGGATAATTAGAAATAATGACCACATCCGTCGG	induced expression of $expR_{Ecz}^{W54A}$
pET28a-W54A-3	CCGACGGATGTGGTCATTATTTCTAATTATCCATCGGAAGCGGTTGAGATTT	

[a]: The lowercase letters in the primers indicate the regions for ligating the amplified DNA to the digested plasmid. The uppercase letters in the primers indicate the regions for DNA amplification. The regions for alanine alteration were underlined on the primers.
